# Availability of synchronous information in an additional sensory modality does not enhance the full body illusion

**DOI:** 10.1007/s00426-020-01396-z

**Published:** 2020-07-27

**Authors:** Lieke M. J. Swinkels, Harm Veling, Ap Dijksterhuis, Hein T. van Schie

**Affiliations:** Behavioural Science Institute, Radboud University, Montessorilaan 3, P.O. Box 9104, 6500 HE Nijmegen, The Netherlands

## Abstract

The Full body illusion (FBI) is an illusion in which participants experience a change in self-location to a body that is perceived from a third-person perspective. The FBI is usually induced through experimenter generated stroking but can also be induced through self-generated stroking. In four experiments (three preregistered) we compared a self-generated stroking induction condition to a self-generated movement condition, where the only difference between conditions was the presence or absence of touch. We investigated whether the illusion reflects an all-or-nothing phenomenon or whether the illusion is influenced by the availability of synchronous information in an additional sensory modality. As a prerequisite, we investigated whether the FBI can also be induced using just self-generated movement in the absence of synchronous touch. Illusion strength was measured through illusion statements. Participants reported an equally strong illusion for both induction methods in Experiments 1, 2 and 3. In the third experiment, we additionally measured the time of illusion onset. Like the illusion strength measures, the illusion onset times did not differ between the two induction methods. In the fourth experiment participants only completed the self-generated movement condition. Again, they reported the FBI, demonstrating that the findings of Experiments 1, 2 and 3 were not dependent on the presence of a condition that used synchronous touch. Together, these findings confirm the hypothesis that the FBI is an all-or-nothing phenomenon and that adding additional multisensory synchronicity does not help to enhance the strength, onset time or onset probability of the illusion.

**Electronic supplementary material:**

The online version of this article (10.1007/s00426-020-01396-z) contains supplementary material, which is available to authorized users.

## Introduction

An important aim in cognitive neuroscience is to understand the basic principles and functional mechanisms that give rise to our embodied sense of self (Blanke, 2012; Blanke & Metzinger, 2009; Ehrsson, 2012; Tsakiris, 2010). Studies on body illusions provide an important contribution to our understanding of embodiment by investigating the conditions in which (illusory) embodiment may or may not occur. It has been shown that body illusions may be induced when two or more sensory modalities (e.g. vision and touch) are activated in a synchronous matter. A well-known example is the rubber hand illusion (RHI) in which participants report ownership over a rubber hand when an experimenter synchronously strokes the (visible) rubber hand and the participants’ (invisible) real hand (Botvinick & Cohen, 1998), but not when stroking is asynchronous and random. Although there is a large body of evidence that multisensory integration between synchronous signals from different modalities is a dominant factor in the construction of the bodily self, contrasting views have emerged with regard to how multisensory integration contributes to embodiment. More specifically, two contrasting ideas have been formulated. Kalckert and Ehrsson (2014) suggest that (illusory) embodiment operates via an all-or-nothing principle. Once a correlation has been established between two sources of information, be it two signals from different sensory modalities, or between signals in sensory and motor cortices during motor control, the inclusion of information from an additional sensory modality will not enhance the degree of illusory embodiment. Conversely, Samad et al. (2015) suggest that illusory embodiment varies as a function of the available sensory evidence for a common cause. According to this account, synchronous activity in an additional sensory modality may strengthen the inference that a body or body part belongs to the self and will increase the strength of the body illusion. The current study aims to resolve these opposing views by investigating the question of whether or not the inclusion of additional synchronous sensory information will enhance the degree of illusory embodiment. The outcomes of the study are relevant for advancing our fundamental understanding of the mechanisms that underlie body ownership (Kilteni et al., 2015), self–location (Blanke, 2012) and construction of the body schema (Maravita et al., 2003) in support of action (de Vignemont, 2010). Insights may hold relevance for the treatment of neuropsychological patients with deficits involving body representations such as phantom limb pain (Moseley et al., 2012; Ramachandran et al., 1995), alien hand syndrome (Schaefer et al., 2013), and xenomelia (Lenggenhager et al., 2015), or may support interventions that facilitate the acceptance and control of artificial limbs following amputation (Ehrsson et al., 2008; Rognini et al., 2019). In addition, the study findings may be relevant for the induction of body illusions in the treatment of chronic pain (Pamment & Aspell, 2017) and the creation of effective methods for inducing first and third person body illusions in virtual reality (Debarba et al., 2015; Galvan Debarba et al., 2017).

The conclusion by Kalckert and Ehrsson (2014) that (illusory) embodiment functions as an all-or-nothing phenomenon reflects the outcomes of their experiments in which they compared different methods to induce the RHI. They found body ownership over the rubber hand to be equally strong with the traditional static induction of the RHI through stroking by an experimenter, an active movement condition in which active movements of the participants’ real hand animated the rubber hand, and a passive movement condition in which the real hand and the rubber hand were moved by the experimenter (Kalckert & Ehrsson, 2014). Comparable effects were found in an earlier study from the same lab that compared active and passive induction of the RHI through movement (Kalckert & Ehrsson, 2012). Furthermore, other labs have reported comparable levels of ownership with different induction methods (Brugada-Ramentol et al., 2019; Pyasik et al., 2019; Riemer et al., 2013).

Nevertheless, several inconsistent findings have been reported which cast doubt on the conclusion that the strength of body illusions is independent of the specific method of induction. For instance, Walsh et al. (2011) found that passively induced synchronous movements led to higher reports of illusory body ownership than movements that were self-induced, whereas Dummer et al. (2009) found a trend towards stronger illusory embodiment of a rubber hand with active induction than with passive induction. Strikingly, Ma and Hommel (2015) found that body ownership was much stronger in a condition in which a virtual hand illusion (VHI) was evoked trough self-movement versus a static condition in which the illusion was evoked by synchronous visuotactile stimulation [also see Ma et al. (2017) for similar results]. These latter findings may be explained by the larger degrees of freedom in virtual reality and the opportunity for participants to acquire a more extensive set of multisensory samples supporting the experience of ownership and agency over the virtual hand.

Altogether, it seems too early to conclude that different methods for inducing illusory embodiment are equally effective and that illusory embodiment operates as an all-or-nothing phenomenon. A complicating factor in this discussion is that comparisons in the strength of body illusions between active, passive and static induction methods may be confounded by differences in the experience of agency during action execution (Gallagher, 2000; Tsakiris et al., 2006, 2007). Although, research on the (in)dependence of agency and body ownership is still ongoing and unresolved, several studies have found increases in agency to be accompanied by increases in body ownership (Brugada-Ramentol et al., 2019; Kalckert & Ehrsson, 2017; Ma & Hommel, 2015; Ma et al., 2017; Tsakiris et al., 2006). Hence, increases in agency in association with active movement might add to the experience of owning an artificial body or body part.

Another problem that potentially invalidates comparisons between illusions evoked by (passive or active) movement and static conditions, is that actions will activate a large number of proprioceptive channels in the skin, muscles, tendons and joints that register changes in limb positions, joint angles, and force applied to the body. Likewise, when movements involve the head or the trunk, vestibular channels that register acceleration and balance (Day & Fitzpatrick, 2005) will become activated. Considering that interactions between sensory channels may result in suppression or enhancement depending on the task and movement (Walsh et al., 2011), effects on body illusions and comparisons between static—and movement induced body illusions may be difficult to predict and come to vary across experiments. In the present study, we circumvented these methodological problems by making a direct comparison between two similar movement conditions in which we only vary the inclusion of the tactile modality.

The hypothesis that the strength of body illusions may vary as a function of the available sensory evidence was forwarded by Samad et al. (2015). The authors adopted a Bayesian causal inference model of multisensory perception to account for the rubber hand illusion (RHI). In their model, a body illusion is characterized by the inference of a common cause for conflicting proprioceptive, visual and tactile sensations. The strength of the overall evidence for a common cause in the end determines the probability that a common cause is inferred and that the illusion arises. Their model predicted that synchronous stroking would strengthen the illusion as compared to a version in which the rubber hand was merely watched and not stroked. This prediction was confirmed in an experiment. The results suggest that the addition of more synchronous visuotactile information strengthened the overall evidence for a common cause, not only tipping the balance to a point where the illusion was experienced but creating a stronger illusion experience as compared to the condition where participants merely watched the rubber hand (Samad et al., 2015).

It is the question, however, whether the experiment by Samad et al. (2015) really proves the idea that an induced body illusion can be strengthened by additional synchronous information. In their study, at least one quarter of all participants scored below the midpoint on the ownership question (Q3) “I feel like the rubber hand is my hand” at the pre-test when presented with the rubber hand. The increase in ownership that was reported after visuotactile stimulation could (at least partly) be explained by this subset of participants who did not report the illusion at the pre-test. Furthermore, it is also possible that participants who scored positively on the ownership question did not experience a full-blown body illusion but simply acknowledged the idea that visual and spatial properties of the hand could pass as their own hand. Without more objective measures of body ownership such as skin conductance in response to threat (Armel & Ramachandran, 2003) or proprioceptive drift (Botvinick & Cohen, 1998) it is difficult to determine which participants did experience a RHI and which did not. Consequently, it could be that the average increase in body ownership in the experiment of Samad et al. (2015) reflects the induction of the RHI in participants who did not experience an illusion at the pre-test.

A study by Choi et al. (2016) may provide additional support for the hypothesis proposed by Samad et al. (2015). They enriched the acquisition phase of the VHI by including tactile and auditory feedback on participants’ actions with a virtual xylophone. Action feedback was found to systematically enhance the strength of the VHI, supporting the hypothesis that adding synchronous multisensory evidence may increase the strength of body illusions. It should be noted though that Riemer et al. (2019) have argued that these effects probably reflect increased emphasis on the goal-directed aspects of the actions. In line with this interpretation, Wen et al. (2016) have found that proprioceptive drift (an implicit measure of body ownership) and body ownership will increase when participants make goal-directed actions towards a virtual object, as compared to intransitive movements. In addition, including sensory information about the effects of actions will enhance the sense of agency that participants experience over their actions (Haggard, 2017), as acknowledged by Wen et al. (2016). Consequently, agency is probably a confounding factor that limits the interpretation of the findings by Choi and Li (2016). In the current study, the possible confound of action effects is circumvented by using repetitive stroking and waving movements in which the sensory consequences of the action are no goal in itself.

In sum, the question whether the inclusion of additional multisensory information can uplift (illusory) embodiment or whether the illusion should be seen as an all-or-nothing phenomenon can, in our view, not be answered clearly yet on the basis of the current literature. In our study we have chosen to investigate this research question in the context of the full body illusion (FBI) as insights into the nature of illusory embodiment may help to optimize systems of virtual reality and possibly prevent costs for the real body that are considered to accompany online role-playing from a third person perspective (3PP; Ganesh et al., 2012; Swinkels et al., 2020). During the experience of a full body illusion (FBI; Lenggenhager et al., 2007) participants experience touch to originate from a body that they see in front of them and report to feel spatially disjoint from their body. Similar to the RHI, the FBI can be induced by an experimenter who provides synchronous visual and tactile stimulation. The illusion is usually accomplished with the help of a camera positioned behind the participant that provides a live video feed of the stroking by the experimenter that is presented to the participant via a head mounted display (HMD). As is the case with the RHI, the illusion breaks if the visual information and tactile sensations are presented asynchronously and/or or in spatially incompatible locations. Recent studies have found that the FBI may also be self-generated through tactile self-stimulation as mediated by a robotic device (Hara et al., 2014) or more simply through stroking their own neck (Swinkels et al., 2020). Importantly, the functional mechanisms that are held responsible for the FBI, e.g. multisensory integration, are considered to play a central role in other bodily illusions such as the RHI as well (Ehrsson, 2012; Metral et al., 2017; Olive & Berthoz, 2012). Hence, the outcomes of the current experiment should be relevant for the majority of bodily illusions that are considered to rely on similar mechanisms.

In the present study, we describe four experiments in which participants self-induced a FBI. Crucially, in one condition the FBI was evoked through movement; that is, participants repeatedly waved their dominant hand back and forth in parallel to the side of their neck, without touching (movement condition); whereas in the other condition, the same movement was made, while touching the neck (stroking condition). The fact that our two conditions only differ in synchronous information in just one sensory modality should enable us to disentangle the two principles in the present study. If body illusions reflect an all-or-nothing phenomenon, we should find that the illusion strength and onset of the FBI should be identical in the two conditions. If on the other hand body illusions are influenced by the availability of synchronous information in an additional sensory modality, we should expect the self-reported strength of the illusion to increase and or the onset time to shorten in the condition where participants are stroking, relative to waving. In the first experiment we explored the hypotheses under investigation by having participants induce the FBI using both self-generated movement and self-generated stroking in a 3PP. The second experiment was intended as a replication of Experiment 1. In the third^1^ experiment we tried to replicate the findings of Experiments 1 and 2 and additionally looked into the illusion onset times. Finally, in the fourth experiment we controlled for transfer effects between both methods of inducing a FBI and examined if self-generated movement would also be effective in inducing a FBI when presented independently from self-generated stroking. Hypotheses, sample sizes and planned analyses were preregistered for Experiments 2, 3 and 4 to create a clear timestamp of the decisions that were made before the experiments were conducted.^2^

## Ethics statement

All experiments in this paper were approved by the ethics committee social science at Radboud University (ECSW2016-2501-368 and ECSW2016-2501-368a) and were conducted in accordance with the declaration of Helsinki. All participants provided written informed consent before the start of the experiment and received course credit or gift vouchers for their participation. Participants only participated in one of the four experiments.

## Experiment 1

In Experiment 1 we investigated whether the inclusion of synchronous information in an additional sensory modality will or will not enhance the degree in which participants experience the FBI. We adopted the HMD set-up as described in Swinkels et al. (2020) in which self-stroking was successfully applied and compared it to a self-generated movement that was similar to the stroking but without touching the skin. In this way, the two induction methods only differed in one level of synchronous sensory information, namely the presence or absence of touch. As, so far, the self-generated FBI has only been reported with the use of tactile stimulation (Hara et al., 2014; Swinkels et al., 2020) our first hypothesis (H1) tested if the FBI would occur in a condition in which participants simply waved their hand back and forth in a 3PP. In accordance with this hypothesis, we predicted that the perception of synchronous movement should result in stronger ratings on self-report illusion questions than a condition in which participants did not see their movements.

In addition to the basic hypothesis that the FBI can be evoked through movement without touch, the following main hypotheses and predictions were investigated. According to the all-or-nothing (AoN) hypothesis (H2a) the stroking and movement conditions should result in an equally strong FBI as extra sensory evidence should have no additional effect. According to the sensory evidence (SE) hypothesis (H2b), the extra sensory evidence in the stroking condition should result in a stronger FBI than the movement condition. Note furthermore that in addition to the two hypotheses outlined here, there is also the theoretical possibility (H2c) that the FBI will be stronger in the movement condition than in the stroking condition. Although based on the literature we had no clear reason to anticipate this outcome, we still noted this outcome as a possibility.

## Method

### Participants

Twenty-four healthy participants (*M*_age_ = 22.0, range = 18–29, 8 males, 3 left-handed) took part in the experiment. Participants had normal or corrected to normal vision (no glasses, contact-lenses were allowed), did not suffer from motion sickness and had no history of neurological or psychiatric illness as established by self-report.

The number of participants in the study was determined with a power analysis. Based on previous studies using self-generated neck-stroking to induce a FBI we expected a strong effect-size (Swinkels et al., 2020). However, because the illusion statements had to be adapted to also make sense in the movement condition, we conservatively adjusted our expectations to a medium effect-size. Power calculations using a repeated-measures ANOVA within factors with one group, four measurements, an alpha error probability of 0.05 and a medium effect size *f*(U) = 0.41, suggested that a sample of 24 participants was adequate to reach a power of 0.80.

### Head-mounted display set-up

**Fig. 1:**
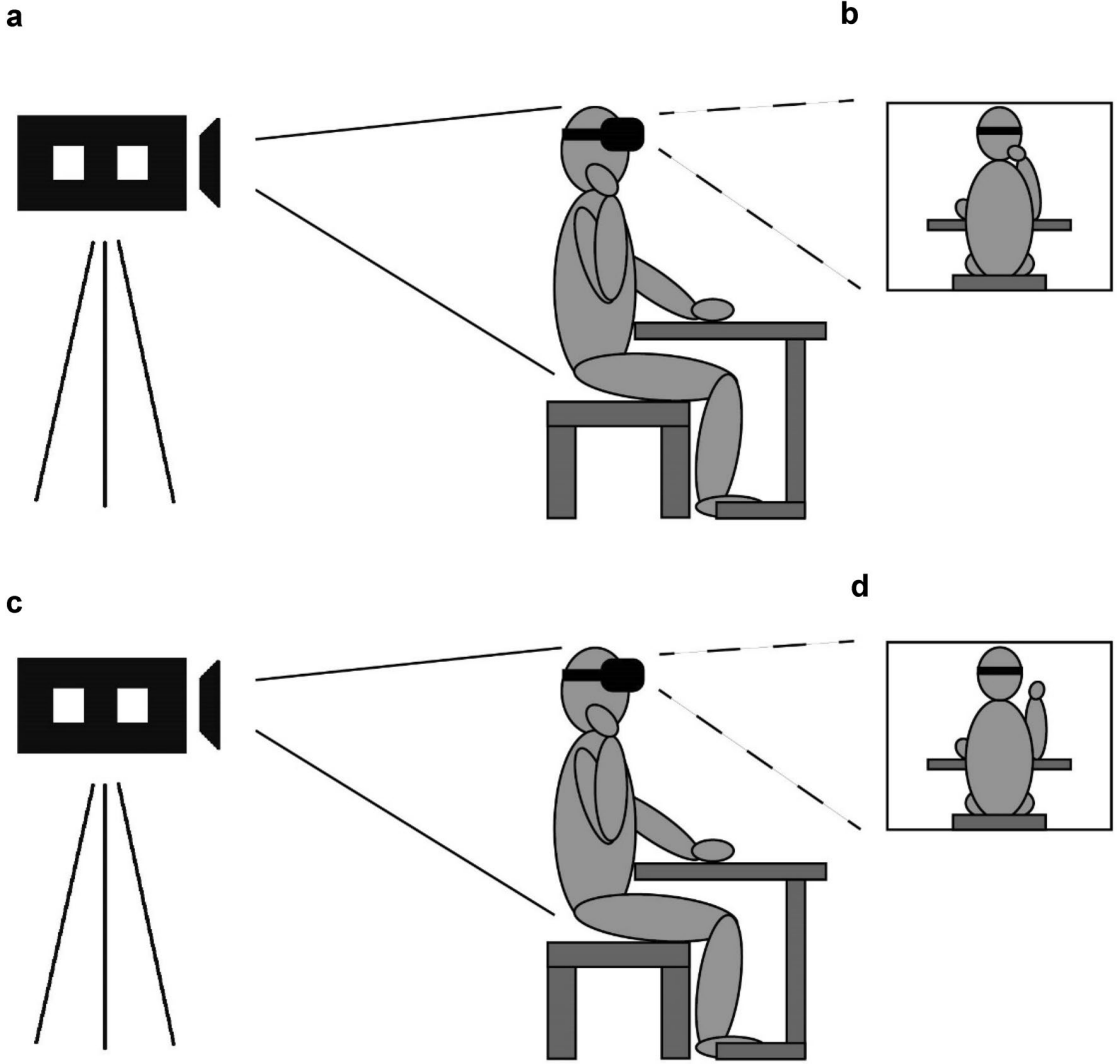
Set-up used in Experiments 1 to 4. **a** and **c** Head-mounted display set-up. **b** The camera image as perceived by the participant on the head-mounted display in the stroking condition. **d** The camera image as perceived by the participant on the head-mounted display in the movement condition

We made use of a head-mounted display (HMD) set-up (Lenggenhager et al., 2007; Swinkels et al., 2020) in which participants were presented with a full-body perspective of their back. A Logitech C920 pro camera (placed on a tripod) was positioned 1.5 m behind the seated participant and filmed the participant. The video image from this camera was projected in real-time onto the HMD (oculus development kit 2, field of view: 90°, display resolution per eye: 960 × 1080, refresh rate: 60 Hz) in the experimental blocks (Figs. 1a and c) using a custom made program called Oculus Camviewer that was run on a Dell Precision T3610 computer. This program can be used to display a live, static or delayed video image on the HMD. The intrinsic delay of the system was approximately 1 frame (~ 33 ms) and was not corrected as this delay is most likely not noticeable to participants (Keetels & Vroomen, 2012). To induce the FBI, participants either stroked their neck with their dominant hand about twice per second (stroking condition, Fig. 1b) or they made a similar movement next to their neck without touching the skin (movement condition, Fig. 1d). The stroking/moving tempo was practiced with a metronome before the illusion induction was started. Participants received corrective instructions from the experimenter in case they deviated too much from the speed that was practiced. Participants could see the back of their moving arm and the rest of their body via the HMD. In the control blocks, a static image of the participant was used (cf. Swinkels et al., 2020). Participants are naturally inclined to synchronise their movements with delayed visual feedback when they make repetitive movements (Normand et al., 2011). As a consequence, participants in one of our pilot studies indicated that they experienced their stroking as synchronous after a while regardless of a variable delay of between 300 and 400 ms. A static control condition circumvents this problem. Multisensory integration between participants’ visual, tactile and proprioceptive sensory perception was disrupted because participants could not see their performed movements, hereby preventing the FBI to occur. Participants did see their own body, as well as their hand either touching the neck or near the neck, keeping everything but the visibility of the movement the same as in the synchronous experimental conditions.

### Illusion statements

For this experiment, we adapted the statements previously used by Swinkels et al. (2020) so that they may be used to measure the FBI in both the stroking and the movement condition (see Table 1). Instead of relocation of touch towards the virtual body our statements now measure a relocation of the proprioceptive experience of the movement the participants make to measure the experienced self-location. In total, the questionnaire consisted of 9 items that were rated on a visual analogue scale^3^ ranging from 0 (“Not at all”) to 100 (“Very strongly”) (for items see Table 1). Participants were asked to indicate to what extent they experienced the statement using the visual analogue scale. The statements were completed after each block and were presented to participants using Inquisit 4 (Millisecond, 2014).

**Table 1:** Illusion statements as used in the four experiments. The first two items were completed in all four experiments. Additional items were completed in Experiments 1 and 3 as indicated in the table. All items were rated on a visual analogue scale ranging from 0 (not at all) to 100 (very strongly)

Main statements Experiments 1, 2, 3 and 4	
S1	At some point, it seemed as if I was feeling the movement of my arm in the location where I saw the arm
S2	At some point, it seemed as though the feeling in my arm was caused by the arm that I saw
Additional statements Experiment 1	
S3	At some point, I felt more in touch with the body in front of me than with my real body
S4	At some point, I felt as if I was drifting towards the body in front of me
S5	At some point, it seemed as if I might have more than one body
S6	At some point, it seemed as though the movement I felt came from somewhere between my real body and the body in front of me
S7	At some point, it appeared (visually) as if the body in front of me was drifting backwards
S8	At some point, it seemed as though I was in two places at the same time
S9	At some point, I felt to be outside of my body
Additional statements Experiment 3	
S3	I felt as if my body was located where I saw the body
C1	I felt my heartrate reduce
C2	I felt my breathing become deeper
C3	I felt my body relax

Statements are shown in the order they were presented to the participants

### Exploratory measures

#### Questionnaires

In addition to the illusion statements, participants completed the short version of the Tellegen Absorption Scale (Tellegen & Atkinson, 1974; for the short version, see: van Elk et al., 2016), the Sensory Suggestibility Scale (Polczyk & Pasek, 2006), some questions regarding their video game habits and the Vividness of Motor Imagery Questionnaire-2 (VMIQ-2; Roberts et al., 2008) as part of a student project. These questionnaires were completed for exploratory purposes and will not be further discussed in this paper.

#### Pressure pain measurement

Previous research indicated that the FBI can be associated with costs for the real body (see also: Swinkels et al., 2020).We therefore wanted to explore the pressure pain threshold as a measure of these costs. A digital algometer (Wagner instruments, FPX 25) was used to measure the pressure pain threshold before and after each movement block. More details can be found in supplementary online materials (SOM1).

#### Procedure

Upon arrival at the lab participants received written and verbal instructions regarding the experiment and provided written informed consent. Participants first completed a demographics questionnaire and the exploratory questionnaires. Next, the participant received specific instructions for the block of the illusion task they were about to complete. For the stroking blocks the participants were instructed to use their dominant hand to stroke the side of their neck in an up- and down fashion while paying attention to the stroking on the HMD. For the movement blocks, the participants received the same instructions but were instructed to put about 10 cm between their moving hand and the skin of their neck.

When the participants had been fitted with the HMD, they were instructed to check whether it provided a sharp image and whether they were sitting in the middle of the image. In the control blocks, a static image of the participant was created before the block started. To create this image, the participant was asked to sit still with their hand either touching the neck (Fig. 1b) or with their hand held at a distance of approximately 10 cm next from the neck (Fig. 1c). The image was recorded by the experimenter with a mouse click. The use of the static image created an asynchrony between the performed movement and the seen movement (absent).

Both the stroking and movement were performed for a duration of four minutes. After four minutes the participants took off the HMD and completed the illusion statements. This procedure was repeated until the participant had completed all four conditions. The conditions were offered in a counterbalanced order with the restriction that participants always completed both the synchronous and the static condition for each induction method (stroking, movement) before moving on to the next. At the end of the experiment, participants were briefly interviewed about their experiences.

#### Statistical analyses

Research on the FBI typically uses the first three items of the (Aspell et al., 2009) illusion questionnaire to determine if a full-body illusion occurred as these items discriminate best between the experimental and the control condition and tap into the most important characteristics of the illusion, namely experienced self-location and body ownership (e.g. Aspell et al., 2009; Lenggenhager et al., 2007; Salomon et al., 2013). We preregistered to focus primarily on the first two items as there is a discussion in the literature about item 3 “I felt as if the body that I saw was my body”, which is often argued to reflect self-recognition in the context of the FBI (e.g. see, Petkova et al., 2011; Pomés & Slater, 2013). Although S3 seems successful at differentiating between the experimental and the control condition in FBI experiments (Aspell et al., 2009; Lenggenhager et al., 2007), the ratings on item 3 in the control condition have been shown to be relatively high (e.g. Salomon et al., 2013), suggesting that item 3 may not only provide a measure of FBI but also of self-recognition. This makes this item less suitable for determining if participants experienced the FBI or not. Based on these considerations we decided to design an alternative third item to gain insight into feelings of embodiment related to the body in front of the participant. The analysis on this item was exploratory but will be shown here for completeness. The other items were only administered for completeness and will not be discussed further in this paper. We conducted a 2 (Induction method: stroking, movement) by 2 (Video condition: synchronous, static) repeated measures ANOVA separately for illusion statement 1 (S1), 2 (S2) and 3 (S3). Additionally, Bayesian paired samples *t* tests were conducted using JASP (JASP Team, 2017) on the difference scores for S1, S2 and S3 that resulted from subtracting the synchronous movement condition from the static movement condition and the synchronous stroking condition from the static stroking condition. We used a default Cauchy prior width of 0.707.

## Results

### Illusion statements—non-preregistered

#### Repeated measures ANOVA

**Fig. 2:**
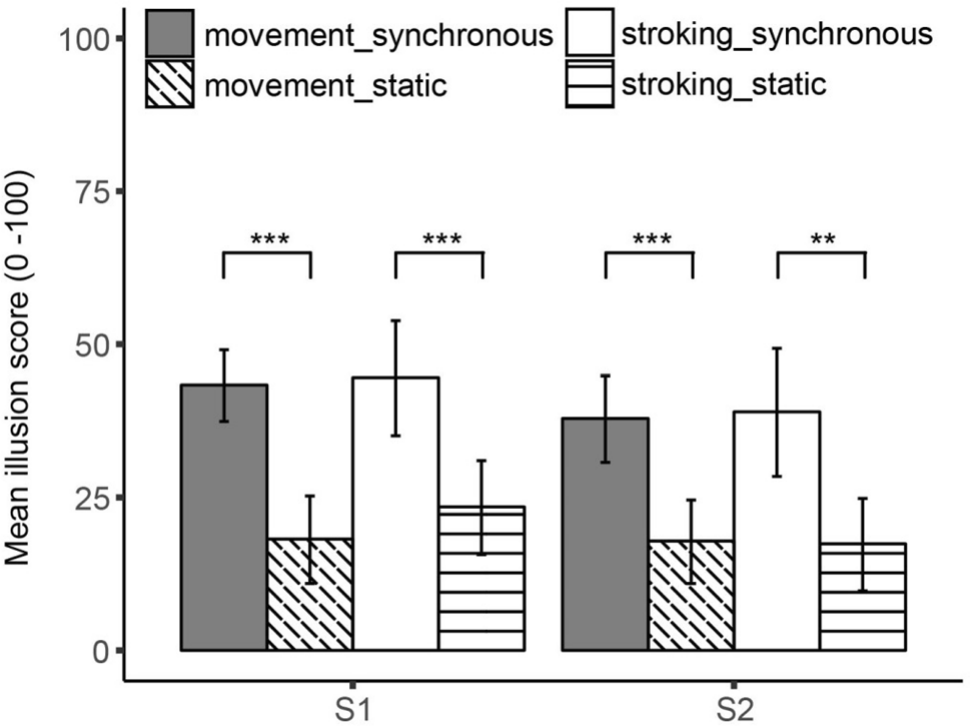
Mean illusion scores obtained for illusion statements 1 and 2 in Experiment 1. Illusion scores are depicted separately for the self-generated movement and the self-generated stroking induction method. Note: *p < 0.05, **p < 0.01, ***p < 0.001. Error bars reflect 95% CI

The repeated measures ANOVA for S1 with induction method and video condition as within-subject factors yielded a significant main effect of video condition, *F*(1, 23) = 26.99, *p* < 0.001, *η*_p_^2^ = 0.54, indicating that participants reported a stronger feeling that they felt the movement of their arm in the location where they saw their arm, after completion of the synchronous experimental blocks than after the static control blocks (see Table 2 for the means). No significant main effect of induction method was obtained, *F*(1, 23) = 0.53, *p* = 0.473, nor a significant interaction effect, *F*(1, 23) = 0.69, *p* = 0.415, indicating that participants reported a stronger illusion in the experimental blocks regardless of the method that was used to induce the illusion, *t*_moving_(23) = 5.85, *p* < 0.001, *d* = 0.99, *t*_stroking_(23) = 3.68, *p* < 0.001, *d* = 0.70 (Fig. 2).

**Table 2:** Mean scores (SD) on the illusion statements of Experiments 1–4 and the control statements (Experiment 3 only). The illusion scores are depicted for the four conditions separately, except for Experiment 4 where no self-generated stroking condition was completed

Statement	Movement sync	Movement stat	Stroking sync	Stroking stat
Experiment 1				
S1	43.38 (28.13)	18.21 (22.04)	44.58 (33.78)	23.46 (26.56)
S2	37.92 (25.45)	17.88 (22.33)	39.00 (32.20)	17.42 (23.54)
S3*	41.29 (29.79)	20.42 (26.91)	34.00 (28.64)	18.00 (20.90)
S4	31.75 (30.08)	20.00 (26.13)	34.12 (28.42)	15.96(18.54)
S5	37.54 (29.80)	15.42 (22.40)	33.54 (31.40)	16.96(21.66)
S6	41.13 (28.44)	23.21 (28.11)	36.75 (31.37)	17.92 (21.58)
S7	22.00 (28.18)	18.54 (25.20)	24.67 (27.77)	13.83 (22.22)
S8	41.63 (32.09)	25.04 (26.97)	37.17 (32.49)	20.83 (24.48)
S9	35.04 (29.67)	16.38 (22.70)	34.63 (27.99)	21.13 (23.63)
Experiment 2				
S1	42.20 (29.98)	16.25 (21.53)	41.80 (29.27)	15.20 (27.72)
S2	34.75 (30.24)	13.90 (24.36)	35.75 (30.40)	16.10 (25.08)
Experiment 3				
S1	56.20 (28.24)	17.65 (20.46)	55.21 (31.77)	24.01 (25.23)
S2	49.06 (28.77)	18.15 (23.44)	50.01 (30.58)	17.55 (21.36)
S3*	46.65 (29.33)	25.45 (24.96)	51.58 (29.17)	29.27 (27.67)
C1	38.51 (24.90)	34.55 (27.07)	41.92 (26.82)	36.25 (25.33)
C2	45.75 (26.56)	46.37 (25.91)	54.66 (27.43)	48.73 (25.35)
C3	51.20 (24.61)	44.15 (25.94)	54.28 (26.49)	47.93 (24.12)
Experiment 4				
S1	41.65 (25.05)	15.53 (17.92)	–	–
S2	35.41 (27.04)	18.00 (23.04)		

*S3 in Experiment 1 and S3 in Experiment 3 refer to different statements. See Table 1

A similar pattern of results was obtained for S2. The repeated measures ANOVA with induction method and video condition as within-subject factors yielded a significant main effect of video condition, *F*(1, 23) = 19.26, *p* < 0.001, *η*_p_^2^ = 0.46, no significant main effect of induction method, *F*(1, 23) = 0.01, *p* = 0.933, and no significant interaction, *F*(1, 23) = 0.04, *p* = 0.847. After completing the synchronous experimental blocks, participants reported a stronger sense that the feeling in their arm was caused by the arm that they saw, than after the static control blocks, *regardless* of the method that was used to induce the illusion, *t*_moving_(23) = 4.90, *p* < 0.001, *d* = 0.85, *t*_stroking_(23) = 2.80, *p* = 0.010, *d* = 0.78 (Table 2, Fig. 2).

The exploratory analysis on S3 again shows a similar pattern, with effects of video condition: *F*(1, 23) = 18.50, *p* < 0.001, *η*_p_^2^ = 0.45, induction method: *F*(1, 23) = 1.57, *p* = 0.223, interaction: *F*(1, 23) = 0.49, *p* = 0.492. Participants felt more in touch with the body they saw in front of them than their real body after completing the synchronous experimental blocks than after the static control blocks, regardless of the method that was used to induce the illusion, *t*_moving_(23) = 3.56, *p* = 0.002, *d* = 0.73, *t*_stroking_(23) = 3.09, *p* = 0.005, *d* = 0.62 (Table 2).

#### Bayesian paired samples *t* test

The Bayes factor for S1 was BF_01_ = 3.41^4^, indicating that the observed data are 3.41 times more likely under the null-hypothesis that postulates no difference in illusion strength between the two induction methods than under the alternative hypothesis that postulates that one induction method leads to a stronger illusion than the other. For S2 we obtained a similar effect, BF_01_ = 4.58. This Bayes factor indicates that the observed data are 4.58 times more likely under the null-hypothesis that postulates no difference in illusion strength between the two induction methods. For S3 we found a Bayes factor of BF_01_ = 3.74, indicating that the observed data are 3.74 times more likely under null-hypothesis that postulates no difference in illusion strength between the two induction methods.

## Discussion

The findings in Experiment 1 confirmed the basic hypothesis that the FBI can be evoked by movement in a 3PP, without touch. Furthermore, and most central to the current study, the results provided clear support for the AoN hypothesis and disconfirm the SE hypothesis. That is, the inclusion of additional touch in the stroking condition did not in any way increase the strength of the FBI. Bayesian tests indicated that the illusion was equivalent in both conditions.

In Experiment 2 we attempted to replicate these findings. Sample-size, hypotheses and planned analyses were preregistered for this experiment.

## Experiment 2

## Methods

The methods for Experiment 2 were very similar to the methods for Experiment 1. Only differences will be described.

### Participants

Twenty-one healthy participants participated in this experiment for gift vouchers or course credit. One participant was already excluded and replaced during data collection due to severe concentration problems. Power calculations using a repeated-measures ANOVA within factors with one group, two measurements, an alpha error probability of 0.05 and the smallest effect size obtained in Experiment 1^5^ (*f*(U) = 1.38), suggested that a sample of 7 participants would have been adequate to reach a power of 0.80. However, since we had an exploratory perceived sweetness task (see SOM2) for which we did not yet know what effect size to expect, we recruited 20 participants for the experiment (*M*_age_ = 23.0, range = 19–30, 5 males, 2 left-handed). In addition to the exclusion criteria mentioned under Experiment 1, participants could not participate in Experiment 2 if they suffered from diabetes.

### Illusion statements

In Experiment 2, participants only completed the first two illusion statements (Table 1).

### Exploratory measures

#### Questionnaires

Participants only completed the VMIQ-2 (Roberts et al., 2008).

#### Perceived sweetness

Participants rated grenadine solutions on sweetness. For more details see the SOM2.

#### Procedure

Upon arrival at the lab, participants were welcomed and received instructions about the experiment. Participants first completed the demographics questionnaire followed by the VMIQ-2. After these questionnaires, they completed a practice block for the perceived sweetness task. Next, they received instructions for the FBI task. These instructions were the same as described for Experiment 1, with one difference: the participants were instructed to leave the HMD on their heads after the four minutes of stroking/waving were completed. After each condition, participants were presented with a drink for the perceived sweetness task. When the tasting was completed, participants could take off the HMD and completed the illusion questionnaire. The FBI task, tasting and illusion questionnaire were repeated for the other three conditions in a counterbalanced order as described for Experiment 1.

## Results

### Illusion statements—preregistered

#### Repeated measures ANOVA

**Fig. 3:**
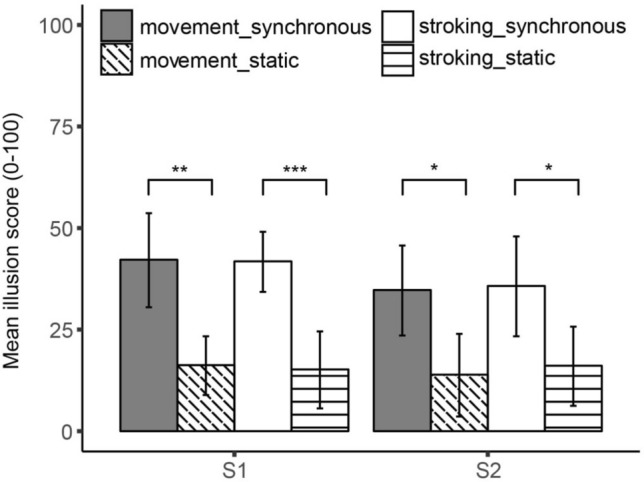
Mean illusion scores obtained for illusion statements 1 and 2 in Experiment 2. Illusion scores are depicted separately for the self-generated movement and the self-generated stroking induction method. Note: *p < 0.05, **p < 0.01, ***p < 0.001. Error bars reflect 95% CI

As in Experiment 1, the repeated measures ANOVA on S1 and S2 with induction method and video condition as within-subject factors yielded a significant main effect of video condition, S1: *F*(1, 19) = 23.10, *p* < 0.001, *η*_p_^2^ = 0.55; S2: *F*(1, 19) = 11.74, *p* = 0.003, *η*_p_^2^ = 0.38 (Table 2, Fig. 3). No significant main effect of induction method was obtained, S1: *F*(1, 19) = 0.03, *p* = 0.868; S2: *F*(1, 19) = 0.07, *p* = 0.791, nor a significant interaction effect, S1: *F*(1, 19) = 0.01, *p* = 0.925; S2: *F*(1, 19) = 0.02, *p* = 0.889, indicating that participants reported a stronger illusion in the experimental blocks *regardless* of the method that was used to induce the illusion, S1: *t*_moving_(19) = 3.92, *p* = 0.001, *d* = 1.00, *t*_stroking_(19) = 4.24, *p* < 0.001, *d* = 0.96; S2: *t*_moving_(19) = 2.80, *p* = 0.011, *d* = 0.78, *t*_stroking_(19) = 2.77, *p* = 0.012, *d* = 0.72 (Table 2, Fig. 3).

#### Bayesian paired samples *t* test

The Bayes factor for S1 was BF_01_ = 4.29 and the Bayes factor for S2 was BF_01_ = 4.27. This indicates that the observed data are respectively 4.29 and 4.27 times more likely under the null-hypothesis that postulates no difference in illusion strength between the two induction methods than under the alternative hypothesis that postulates that one induction method leads to a stronger illusion than the other.

## Discussion

The results of this pre-registered experiment corroborated the findings of Experiment 1. Self-movement without touch was found sufficient to induce a FBI. Furthermore, and importantly, the FBI was found to be equally strong in both conditions as indicated by Bayesian statistics, corroborating the AoN hypothesis, and disconfirming the SE hypothesis.

In Experiment 3, additional evidence is sought to distinguish between the two main hypotheses of interest (AoN hypothesis and the SE hypothesis) by investigating the time of onset of the FBI.

## Experiment 3

In our two experiments so far, we did not find that the inclusion of synchronous touch on top of the synchronicity that accompanies movement execution, enhances the FBI. This suggests that the FBI is not sensitive to extra evidence of sensory synchronicity and that the (illusory) embodiment operates in accordance with the all-or-nothing principle. However, it may be the case that additional synchronous sensory information may contribute to the speed of illusion onset. From the perspective of the SE hypothesis, it could be that additional sensory evidence supporting a common (illusory) origin would cause the critical threshold for the body illusion to be reached sooner than a condition in which this additional sensory evidence is not available. In other words, it may be that extra sources of synchronicity—like touch in our experiments—do not add to the strength of the illusion once that threshold is met, but provide additional sensory evidence that may influence the speed or the probability of the illusion to come about. In Experiment 3 we asked participants to indicate the time at which they felt the onset of the FBI (cf. Kalckert & Ehrsson, 2017) to test this possibility.

The following main hypotheses and predictions were investigated. According to the AoN hypothesis (H1), the stroking and movement methods should be equally fast in inducing a FBI, as extra sensory evidence should have no additional effect. According to the SE (H2) the extra sensory evidence in the stroking condition should result in a faster onset of the FBI than the movement condition. In addition to these hypotheses, we again investigated the strength of the FBI to determine if the results of our previous experiments would replicate.

## Methods

The methods for Experiment 3 were very similar to the methods of Experiments 1 and 2. Only the differences will be described.

### Participants

A total of 76 participants took part in the experiment (*M*_age_ = 22.1, range = 18–28, 20 males, 1 unknown, 3 left-handed). To determine our sample size, we made use of the PANGEA app (v2.0) by Jake Westfall (jakewestfall.org/pangea/) which is suitable for calculating the power for designs that make use of linear mixed-effects models. Our design consisted of the factor participants (random) and the factor induction method with two levels (stroking, movement). We used an expected effect size d of 0.56 (based on Kalckert & Ehrsson, 2017), two replicates (two measurements of onset time per induction method), 32 as our sample size and the default values for var(error) 0.5 and var(P*I) 0.167. For a justification of these default values see Westfall (2016). The power calculation indicated that this should result in a power of 0.81. A cautious interpretation from the results of our second study suggested that only 35% of the participants reports the illusion for both methods (see also SOM3). For this reason, we ended up testing 76 participants in total to arrive at 32 participants for whom illusion onset can be measured for both induction methods.

Five participants were excluded due to misunderstandings of the questionnaire (e.g. scoring high on one or more of the illusion statements but reporting not to have had the experiences during the illusion check, see below).

### Head-mounted display set-up

In addition to the regular illusion blocks, we added four practice blocks to familiarize participants with the experiences they may have during the illusion task. We had one practice block for each combination of induction method and video condition. The practice blocks preceded the test blocks of each induction method and were not followed by the illusion or control statements. The practice blocks were presented in the same counterbalanced order as the test blocks that followed.

### Illusion statements

In addition to the two statements that were administered in Experiment 3, participants completed an additional statement regarding the experienced location of their body in the current experiment (see Table 1). This statement was chosen as an alternative to S3 in Experiment 1 because we wanted to have an additional test of the degree to which participants identified with the virtual body and this alternative item had been used successfully in previous research by other groups (Debarba et al., 2015; Galvan Debarba et al., 2017; Kokkinara & Slater, 2014).

### Control statements

In addition to the three illusion statements we also added three control statements to control for the possibility that some participants may be inclined to answer affirmatively to any question on bodily experience (see Table 1).

### Illusion check

An illusion check was performed in which the scores on the illusion statements were inspected and the participants were asked about their experiences. The goal of this check was twofold: (1) the check was performed to determine whether participants’ interpretation of the experience corresponded with the way they used the illusion scale and answered the illusion statements and (2) to check which participants affirmed experiencing the illusion. The check was performed for each induction method separately after both the practice and the test blocks had been completed and the illusion statements had been rated. First the illusion scores were inspected. Next, in a semi-structured interview participants were asked to describe their experiences, to indicate whether they believed an illusion occurred and to indicate whether the illusion was stronger for the condition with a moving video image or the condition with a static video image. If they expressed an experience that did not correspond with the way they scored it on the illusion statements they were asked further questions to clarify the discrepancy. The following criteria were used to determine if a participant experienced the illusion: (1) participants had a higher average score on the illusion statements in the synchronous condition compared to the static control condition for this method and (2) the interview confirmed that the participant experienced the full-body illusion for this method. This resulted in a binary outcome variable for illusion onset which indicated for each participant whether the illusion was experienced for that induction method or not.

### Illusion onset

To gain insight into the temporal development of the illusion and potential differences between the two induction methods, the illusion onset time was measured. Illusion onset was only measured for participants who affirmed experiencing the illusion according to the criteria described under illusion check. To log the time of illusion onset, participants completed the synchronous condition again with the same induction method as in the test block they had just completed. They were instructed to signal the experimenter the moment at which they first started experiencing the illusion again. The experimenter then logged the time of illusion onset. This procedure was repeated once more for a better estimate of the illusion onset time (Kalckert & Ehrsson, 2017; Metral et al., 2017).

### Exploratory measures

Two questionnaires were completed for exploratory purposes as part of a student project. Empathy was measured with the Toronto Empathy Questionnaire (Spreng et al., 2009). Fantasy proneness was measured with the Creative Experiences Questionnaire (Merckelbach et al., 2001). The questionnaires were administered at the end of the experiment and will not be further discussed.

### Procedure

In Experiment 3 the illusion tasks for each induction method consisted of (1) Two practice blocks, one for the synchronous and one for the static condition, (2) two test blocks, one for the synchronous and one for the static condition, each followed by the illusion and control statements, (3) an illusion check in which the scores on the illusion statements were inspected and the participants were asked about their experiences and (4) an illusion onset measurement (optional) that was only completed in case the participants met the criteria described under illusion onset.

Participants were instructed to take off the headset after completion of each block and to take some time to stretch their arm before moving on to the next block. This allowed a potential illusion to subside before the next block commenced, making sure that there were as little carry-over effects as possible. Instead of four minutes, each block only lasted three minutes in Experiment 3 to minimize the load for participants. This decision was based on previous research in our lab in which we found that it takes up to 96.3 ± 69.3 s on average to induce the illusion. This is still well under three minutes. The experiment ended with the exploratory questionnaires and a demographics questionnaire.

### Statistical analyses

To analyse the illusion statements we first used separate linear mixed-effects models^6^ using the lmer function of the lme4 package (version 1.1.17; Bates et al., 2015) in R (R Core Team, 2015). Our model included a fixed intercept and a fixed effect for the factors induction method (stroking, moving), video condition (synchronous, static) and their interactions (all coded using sum-to-zero contrasts). The repeated measures nature of the data was modelled by including a per-participant random adjustment to the fixed intercept (“random intercept”). To determine p-values we computed Type 3 bootstrapped likelihood ratio tests (using 1000 simulations) as implemented in the mixed function of the package afex (Singmann et al., 2017), which in turn calls the function PBmodcomp of the package pbkrtest (Halekoh & Højsgaard, 2014). To explore potential suggestibility effects, the same model was used for the separate control statements. To further explore the potential suggestibility effects, we conducted an additional linear mixed-effects model. This model included the additional factor statement type (illusion, control) and its interactions with the factors induction method and video condition.

To analyse the illusion onset times, we made use of the same procedure as was used for the analysis of the statements. The model included a fixed intercept and a fixed effect for the factor induction method (stroking, moving; coded using sum-to-zero contrasts) and a per-participant random adjustment to the fixed intercept. The factor video condition was redundant as the onset times were only measured in the synchronous condition and was, therefore, left out of the model. The analysis was conducted on the 32 participants who reported the illusion for both induction methods.

Additionally, Bayesian paired samples t-tests were conducted using JASP (JASP Team, 2017) on the difference scores for S1, S2 and S3 as described for Experiment 1 and on the average onset times of the two induction methods. We used a default Cauchy prior width of 0.707.

## Results

### Illusion statements

**Fig. 4:**
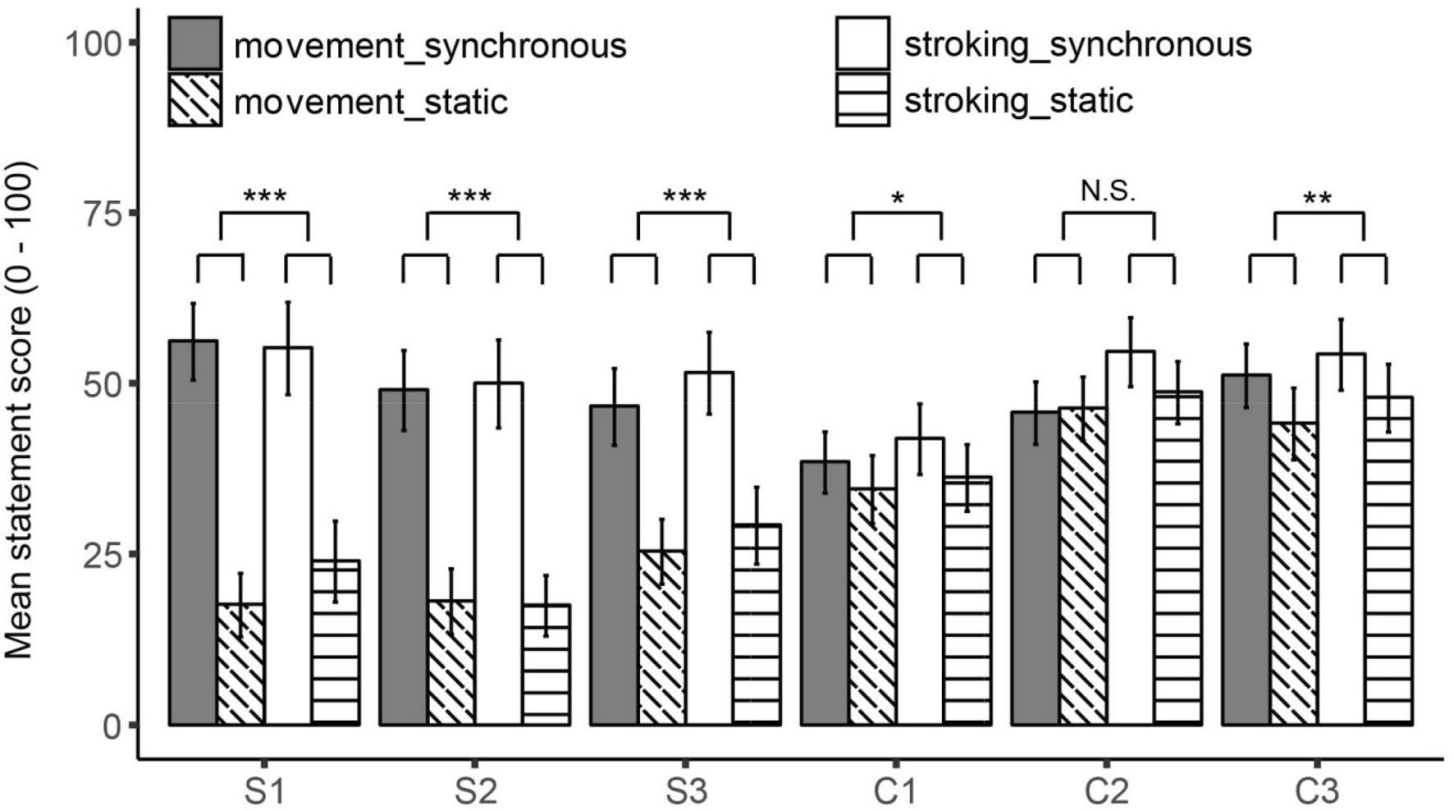
Mean statement scores obtained for illusion statements 1,2 and 3 and control statements 1,2 and 3 that were used in Experiment 3. Statement scores are depicted separately for the self-generated movement and the self-generated stroking induction method. Note: *p < 0.05, **p < 0.01, ***p < 0.001. Stars reflect the significant main effects of video condition. Error bars reflect 95% CI

The separate linear mixed model analyses resulted in a significant main effect of video condition for all three statements, Estimate_s1_ = − 17.44 (1.31), PBtest = 130.45, *p* < 0.001, Estimate_s2_ = − 15.84 (1.26), PBtest = 119.16, *p* < 0.001 and Estimate_s3_ = − 10.88 (1.12), PBtest = 79.42, *p* < 0.001. This indicates that after the synchronous experiment blocks participants: (1) reported a stronger feeling that they felt the movement of their arm in the location where they saw their arm, (2) reported a stronger sense that the feeling in their arm was caused by the arm that they saw and (3) reported a stronger sense that they were feeling their body in the location where they saw the body (see Table 2 for the means) as compared to the static blocks. No significant main effect of induction method was found for any of the three statements, Estimate_s1_ = − 1.35 (1.31), PBtest = 1.07, *p* = 0.293, Estimate_s2_ = − 0.09 (1.26), PBtest = 0.005, *p* = 0.937 and Estimate_s3_ = − 2.19 (1.12), PBtest = 3.86, *p* = 0.063. Nor did we obtain any significant interaction effects, Estimate_s1_ = − 1.84 (1.31), PBtest = 1.99, *p* = 0.175, Estimate_s2_ = − 0.39 (1.26), PBtest = 0.10, *p* = 0.760 and Estimate_s3_ = 0.28 (1.12), PBtest = 0.06, *p* = 0.831, replicating the results of Experiments 1 and 2. Taken together, these results indicate that participants experienced a stronger illusion in the experimental synchronous blocks, regardless of the method that was used to induce the illusion (Table 2, Fig. 4).

### Control statements

The separate linear mixed model analyses resulted in a significant main effect of video condition for statement C1 and C3, Estimate_c1_ = − 2.40 (0.86), PBtest = 7.79, *p* = 0.012 and Estimate_c3_ = − 3.35 (1.06), PBtest = 9.97, *p* = 0.003. This indicates that participants felt their heartrate reduce more (C1) and their body relax more (C3) after the synchronous conditions compared to the static conditions. For C2 no significant main effect of video condition was obtained, Estimate_c2_ = − 1.33 (1.00), PBtest = 1.79, *p* = 0.204. The main effect of induction method was only significant for C2, Estimate_c2_ = − 2.82 (1.00), PBtest = 7.95, *p* = 0.004, and not for C1 or C3, Estimate_c1_ = − 1.28 (0.86), PBtest = 2.23, *p* = 0.132 and Estimate_c3_ = − 1.71 (1.06), PBtest = 2.66, *p* = 0.104. None of the interaction effects were significant, Estimate_c1_ = 0.43 (0.86), PBtest = 0.25, *p* = 0.625, Estimate_c2_ = 1.64 (1.00), PBtest = 2.71, *p* = 0.097 and Estimate_c3_ = − 0.17 (1.06), PBtest = 0.03, *p* = 0.887 (Table 2, Fig. 4).

### Exploratory Linear mixed model analysis on combined statements

The fact that we also obtained significant main effects of video condition for two of the control statements suggests that we may be dealing with suggestibility effects. However, a closer look at Fig. 4 shows that the effect of video condition may be different for the control statements than for the illusion statements. To further explore this possibility, we also conducted an exploratory linear mixed model analysis on the combined statements as described under statistical analyses. This analysis resulted in a significant main effect of induction method, Estimate = − 1.57 (0.55), PBtest = 8.27, *p* = 0.002, indicating that participants gave higher scores to the statements after completion of the stroking method (M = 42.62, SD = 29.47) than after completion of the movement method (M = 39.47, SD = 28.58). We also obtained a significant main effect of statement type, Estimate = 4.31 (0.55), PBtest = 61.25, *p* < 0.001, indicating that participants gave higher scores on the control statements (M = 45.36, SD = 26.50) compared to the illusion statements (*M* = 36.73, *SD* = 30.84). We also found a significant main effect of video condition, Estimate = − 8.54(0.55), PBtest = 227.99, *p* < 0.001, indicating that on average higher scores were given to the statements after completion of the synchronous conditions (M = 49.58, SD = 28.28) compared to the static conditions (M = 32.51, SD = 27.28). Most importantly, we found a significant interaction between video condition and statement type, Estimate = 6.18(0.55), PBtest = 123.33, *p* < 0.001, indicating that the effect of video condition is not the same for the illusion statements and the control statements (see Fig. 4). For the illusion statements, the differences between the synchronous and the static conditions were much larger than for the control statements. Additionally, the average scores obtained for the illusion statements in the static conditions indicate that participants most likely did not experience these illusory experiences or very weakly as compared to the synchronous conditions. However, the average scores obtained for the control statements in the static conditions indicate that participants most likely did have the control experiences to an almost similar extent as in the synchronous conditions. None of the other interactions were significant.

#### Bayesian paired samples *t* test

The Bayesian paired samples t-test on the full dataset yielded the following results: The Bayes factor for S1 was BF_01_ = 1.92, the Bayes factor for S2 was BF_01_ = 7.15 and the Bayes factor for S3 was BF_01_ = 7.38. This indicates that the observed data are respectively 1.92, 7.15 and 7.38 times more likely under the null-hypothesis that postulates no difference in illusion strength between the two induction methods than under the alternative hypothesis that postulates that one induction method leads to a stronger illusion than the other. When only participants who reported an illusion for both conditions were included the Bayes factors were BF_01_ = 4.39, BF_01_ = 2.19 and BF_01_ = 5.29 respectively for S1, S2 and S3.

#### Onset times

**Fig. 5:**
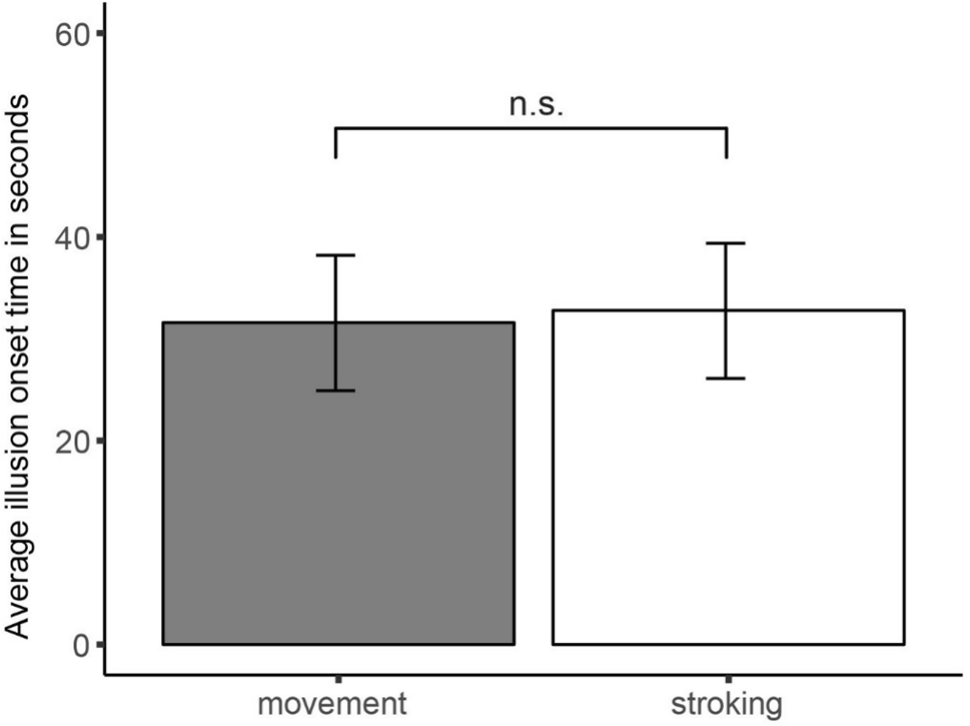
The mean illusion onset times depicted for the self-generated movement and the self-generated stroking condition. Note*:* Error bars reflect the 95% CI

The average onset time in the self-generated movement condition was 31.6 s (SD = 31.9), the average onset time in the self-generated stroking condition was 32.8 s (SD = 26.2) (See Fig. 5). For both induction methods, 95% of the participants who report the illusion reported it within 95 s (self-generated movement condition: 94.7 s, self-generated stroking condition: 95 s).

The linear mixed models analysis on the onset times indicated that there was no significant difference between the two induction methods in the time it took for the illusion to arise, Estimate = − 0.59 (1.82), PBtest = 0.11, *p* = 0.777. The onset times of the two induction methods showed a positive correlation, *r*(31) = 0.53, *p* = 0.002. One participant did not report the illusion the second time it was measured in the moving condition and was assigned the maximum trial duration of 180 s (Metral et al., 2017). Removing this participant did not change the results, Estimate = -1.76(1.59), PBtest = 1.22, *p* = 0.274, nor did it change the correlation, *r*(30) = 0.54, *p* = 0.002.

#### Probability of the illusion

Finally, we explored whether participants would be more likely to report experiencing theillusion for one of the two induction methods.^7^ Of the final number of 71 participants, 32 reported a FBI for both induction methods (~ 45%). Seven participants reported a FBI for the self-generated stroking condition only (~ 10%), 16 participants reported a FBI for the self-generated movement condition only (~ 23%) and 16 participants did not experience the illusion for either of the two induction methods (~ 23%). This means that ~ 77% of the participants experiences the illusion for at least one of the methods. We first tried to run a generalized linear mixed model with the binary outcome variable illusion onset (yes, no), a fixed intercept, a random intercept for participant and induction method as a fixed factor. However, even though the model did converge, the type 3 bootstrapped LRT would not converge without warnings meaning that we could not get reliable *p* values, not even when the number of iterations was maximised or when a different optimizer was used. We, therefore, conducted a RM logistic regression analysis in SPSS (v25; IBM SPSS Statistics, 2017) instead. This analysis indicated that there was no significant difference between the times at which participants reported the onset of the illusion in the stroking condition and in the movement condition, OR = 0.70, 95% CI = [− 0.11, 0.94], Wald Chi-square = 2.38, *p* = 0.123. Additionally computed confidence intervals for the generalized linear mixed model show that the confidence interval for the induction method crosses zero, suggesting that the probability of reporting the illusion does not differ for the onset models. This latter analysis confirms the outcomes of the mixed function and the RM logistic regression in SPSS.

#### Bayesian paired samples *t* test

The Bayes factor for the average onset times was BF_01_ = 5.13, indicating that the observed data are 5.13 times more likely under the null-hypothesis that postulates no difference in illusion onset time between the two induction methods than under the alternative hypothesis that postulates that one induction method leads to a faster illusion onset than the other.

## Discussion

In the third experiment, we replicated the findings of Experiments 1 and 2. Again, we found that the illusion strength was equal for both induction methods. Furthermore, a similar effect was found in the group of participants who reliably reported the FBI with both induction methods. This latter result further corroborates the AoN hypothesis that body illusions operate as an all-or-nothing phenomenon whereby synchronous activity in an additional modality does not further enhance the strength of the illusion. More precisely, by selecting participants who experience the illusion in both respective conditions we can unambiguously show that inclusion of an additional synchronous modality on top of a confirmed illusion does not further deepen the illusion experience.

Importantly, the findings in Experiment 3 also showed that the onset times of the FBI were identical with both induction methods. This rules out the possibility as suggested by the SE hypothesis that adding an additional sensory modality will increase the speed at which evidence for an alternative common cause is collected such that a switch in self-location is experienced at an earlier time. Instead, the finding in this experiment suggests that not so much the amount of synchronous activation is what is driving the speed of onset of body illusions, but rather the length of time in which sensory and/or motor signals are found to be synchronised (Kokkinara & Slater, 2014). The finding that the onset speed of the FBI is not influenced by the method of induction is furthermore corroborated by the analysis of the number of participants who experienced the illusion with either induction method. Analysis of the probability of the illusion indicated that there was no significant difference in the amount of participants who experienced a FBI as a consequence of self-movement or self-stroking in a 3PP. This latter finding suggests that both methods were similarly potent in inducing the FBI.

In Experiment 3 we also investigated the possibility that the FBI as measured with the questionnaire items could reflect enhanced suggestibility or social desirability of participants. To this end, three control items were included that inquired about body perceptions on which no effects were expected. The fact that we found a significant main effect of video condition on two of the control statements suggests that indeed some suggestibility or social desirability was present in our sample. However, a significant video condition * statement type interaction indicated that the effects on the FBI items were much larger than the effects on the control items, ruling out suggestibility as an explanation for the FBI as reported in the current study.

It does seem like we obtained a somewhat stronger illusion in Experiment 3 compared to the previous experiments. Due to the practice sessions, participants may have felt more confident in what they experienced and what not and may have scored their experiences higher than they would have done without the practice.

In the final experiment, we moved attention away from the comparison between the two induction methods and focussed more selectively on the method in which we used active self-generated movements to induce the FBI.

## Experiment 4

The experiments presented thus far are the first to show that a FBI can be self-induced when participants observe their movements in a 3PP. However, even though the FBI was reliably evoked in Experiments 1, 2 and 3, it may still be the case that these findings were due to transfer effects from one induction method to the other. An example of this may, for instance, be found in work by Hohwy and Paton (2010), who demonstrated that participants no longer experienced an illusion of touch on a non-hand object when the basic rubber hand illusion was not induced beforehand. To test whether the movement-induced FBI can be generated in isolation, without transfer from the stroking condition, we only administered self-movement as an induction method in Experiment 4. We hypothesised that self-movement in a 3PP is sufficient to generate the FBI, even in the absence of the self-stroking condition. The hypothesis, sample-size and planned analyses were preregistered.

## Methods

The methods for Experiment 4 were very similar to the methods of Experiment 2, again only differences will be described.

### Participants

Fifty-eight healthy participants took part in this experiment for gift vouchers or course credit. The sample size calculation was based on the effect size obtained for the perceived sweetness in Experiment 2. Power calculations using a RM ANOVA within factors with one group, two measurements, an alpha error probability of 0.05 and an effect size *f*(U) = 0.41, suggest that a sample of 50 participants is adequate to reach a power of 0.80.

Eight participants had to be excluded during data collection, so data collection was continued until we had our intended sample of fifty participants (*M*_age_ = 23.0, range = 18–30, 18 males, 9 left-handed). One participant did not complete the experiment due to nausea and seven participants were excluded and replaced because of problems related to the high temperatures (> 27 °C) in the test room (i.e. participants became dizzy, found it difficult to focus or their perspiration caused problems with their ability to see through the HMD).

### HMD set-up

In Experiment 4, participants only completed the self-movement condition. Both the experimental synchronous and the static control condition were completed twice in a counterbalanced order with the exception that the next block could not have the same video condition as the preceding block.

#### Perceived sweetness

For more details see SOM2.

#### Exploratory measures

No exploratory measures were included in this experiment.

#### Procedure

The same procedure as in Experiment 2 was followed, with the exception that the self-generated stroking condition was replaced with an extra repetition of the self-generated movement condition.

#### Statistical analysis

Wilcoxon signed-rank tests were conducted on the mean illusion scores for illusion statement 1 and 2 separately because the data were not normally distributed.

## Results

### Illusion statements—preregistered

**Fig. 6:**
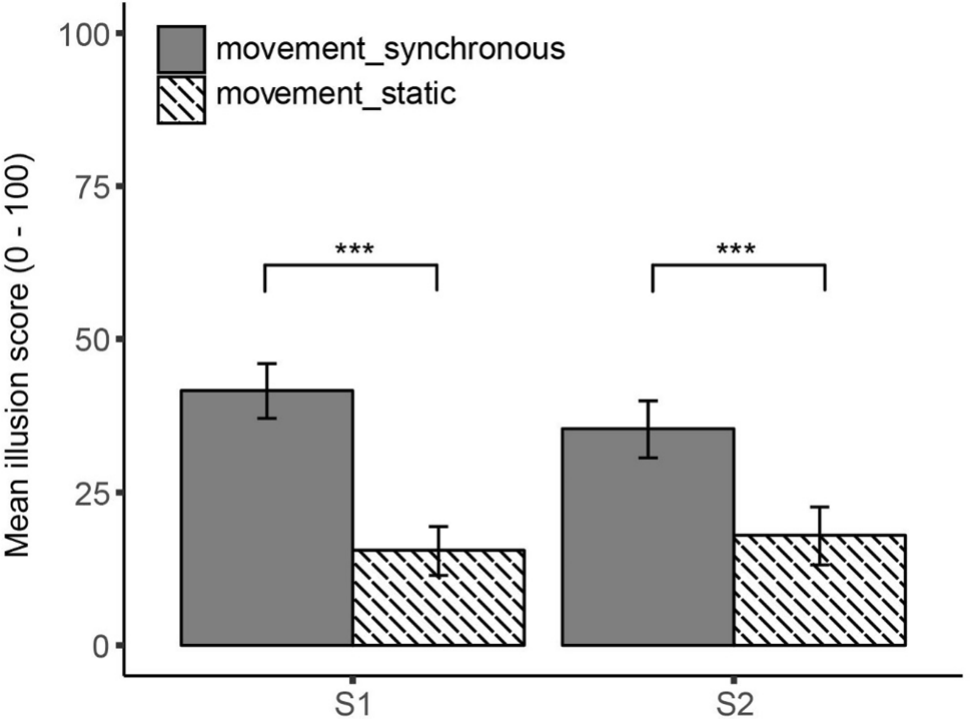
Mean illusion scores obtained for illusion statements 1 and 2 in Experiment 4. Note: *p < 0.05, **p < 0.01, ***p < 0.001. Error bars reflect 95% CI

The Wilcoxon signed rank test yielded a significant difference between the synchronous and the static condition for both S1, *Z* = − 5.61, *p* < 0.001 and S2, *Z* = − 5.04, *p* < 0.001 (Table 2, Fig. 6).

## Discussion

In the fourth experiment we again demonstrated that self-movement can be used to induce a FBI in 3PP. This time, the effectiveness of self-movement as a method was demonstrated in the absence of a self-stroking condition, suggesting that the previous findings were most likely not due to a transfer effect from one induction method to the other.

## General discussion

In the present paper, we investigated the basic functional mechanisms that are responsible for the construction of (illusory) body representations. More specifically, we asked the question whether the availability of synchronous information in an additional sensory modality would increase the strength of (illusory) embodiment or shorten the time at which (illusory) embodiment sets in. Two opposing perspectives were contrasted. The AoN perspective (Kalckert & Ehrsson, 2014) suggests that the (illusory) embodiment functions as an all-or-nothing phenomenon and predicts that adding synchronous sensory information will not enhance the illusion strength or facilitate the onset of the illusion. Oppositely, the SE perspective (Samad et al., 2015) proposes that illusory embodiment varies as a function of the available sensory evidence and predicts that the illusion strength will be enhanced and the time of onset will be shortened with additional synchronous input. In three experiments, we contrasted both perspectives by comparing FBI ratings between two illusion induction methods, one in which the FBI was induced by asking participants to stroke the side of their neck, and another method in which participants were asked to execute the same action while maintaining a 10 cm distance between their hand and neck. Results of the first three experiments unambiguously supported the AoN hypothesis and disconfirmed the SE hypothesis, by revealing that both induction methods were equally strong, equally fast and equally potent in inducing a FBI. In short, the inclusion of extra synchronous tactile information does not have any stimulating effect on the illusion. In Experiment 2, we furthermore confirmed that the FBI can be induced through the perception of simple movements in a 3PP, and that this outcome does not depend on transfer effects from the induction method that included touch.

The current findings provide clear support for the idea that adding synchronous information from an extra sensory modality does not further enhance the level or onset speed of the illusion. The findings in Experiments 1, 2, and 3 matched the predictions of the AoN hypothesis which states that synchrony between a minimum of two (sensory or motor) channels rather than the sum of synchronous information in multiple channels is what is driving (illusory) embodiment. Although the current results are straightforward when it comes to the ineffectiveness of including additional synchronous sensory evidence for illusory embodiment, it is still the question if this also implies that body illusions indeed function as an all-or-nothing phenomenon in the broader sense.

Importantly, and as outlined in the introduction, previous studies have compared ratings of body illusions that were induced by active movements, passive movements and static induction methods (Brugada-Ramentol et al., 2019; Dummer et al., 2009; Kalckert & Ehrsson, 2012, 2014; Ma & Hommel, 2015; Pyasik et al., 2019; Riemer et al., 2013). Some of these studies have found illusion ratings to increase when the illusion was induced via active movements by the participants (Dummer et al., 2009; Ma & Hommel, 2015). These findings clearly conflict with the AoN principle. As pointed out, actively induced body illusions are typically accompanied by a stronger sense of agency, which in turn could have influenced body ownership ratings. It is currently unclear whether agency is to be considered a natural factor in body ownership as has been suggested by (Ma & Hommel, 2015) or if body ownership and agency represent two independent psychological functions (Kalckert & Ehrsson, 2014) that could potentially interact or influence each other. Further research is necessary to disentangle the relationship between agency and body ownership and to determine if the strength of a body illusion (e.g. as reflected in proprioceptive drift, or the location at which participants report to feel their body) can be estimated independently from the sense of agency.

Furthermore, future studies should not only report condition averages but should also pay attention to (the distribution) of individual data points. It could, for instance, be the case that condition effects (e.g. active vs. static induction) are driven by individual participants who do not experience an illusion in the one condition (e.g. in the static condition) and do experience the illusion in another condition (e.g. the active condition). The consequence would be that on a group level it would appear as if the illusion increases in strength, whereas in reality, there is simply a larger number of participants who experience the illusion in the condition with the active induction. Indirect support for this suggestion is presented in SOM3, where we show that the distribution of FBI ratings follows a bimodal distribution which suggests that the average scores on the illusion statements reflect the middle ground between individuals who do experience and individuals who do not experience the illusion. This also explains the relatively low illusion ratings in our experiments. In sum, the current data indicate that additional synchronous evidence does not enhance the strength of body illusions which is consistent with the hypothesis that (illusory) embodiment functions as an all-or-nothing phenomenon. We do realize, however, that more research is necessary to clarify the inconsistent findings that have appeared in the literature. Part of the solution may be to pay closer attention to the data of individual participants and the distribution of data points that make up group averages.

A prerequisite for addressing the main question of the current study was that the self-movement (waving) condition would be effective in inducing a FBI. In line with previous studies that successfully managed to induce body illusions such as the RHI, the VHI, and full-body ownership illusions from a first-person perspective (1PP) using active movements by the participant (Debarba et al., 2015; Galvan Debarba et al., 2017; Gorisse et al., 2017; Kalckert & Ehrsson, 2012, 2014, 2017; Rognini et al., 2013; Romano et al., 2015; Sanchez-Vives et al., 2010; Walsh et al., 2011), the present study (to the best of our knowledge) is the first to demonstrate that a FBI can be induced by simply observing self-generated movements from a 3PP. We consistently found that the FBI was evoked by self-movement in three consecutive experiments. Results of the fourth experiment furthermore indicated that the self-movement FBI was effective on its own and does not depend on transfer effects (Hohwy & Paton, 2010). This finding goes beyond recent studies that have reported that the FBI can be self-generated through tactile self-stimulation (Hara et al., 2014; Swinkels et al., 2020). More precisely, our findings demonstrate that touch is not a necessary element to induce the FBI, and that the illusion can be induced just as effectively through self-movement.

The current findings indicate that the perception of a self-generated movement in 3PP suffices to induce a FBI and that addition of a tactile component does not offer any advantage with regard to the strength of the body illusion, the speed of illusion onset or the likelihood that the illusion will be induced. These findings may be relevant for developers of virtual role-playing applications that aim to induce illusory embodiment of a virtual 3PP avatar and embodied presence in the virtual environment. We believe that our findings may easily translate to virtual reality applications considering that several body illusions have already been successfully induced in virtual reality (Kilteni et al., 2015). Furthermore, motion capture and via-point animation of avatars may offer a more natural (Slater et al., 2009) and feasible approach to induce virtual embodiment (Spanlang et al., 2014) than continuous self-stimulation through stroking. Considering that self-movement appears to be similarly effective as self-stroking to induce the FBI, we recommend the former approach. Notably, this should not mean that the development of sensory feedback devices should be abandoned (Tactical Haptics, 2017). Considering that action-effect feedback of goal-directed actions has been found to be effective in enhancing agency and illusory ownership (Choi et al., 2016; Riemer et al., 2019; Wen et al., 2016), further development of sensory feedback devices supporting action-effects may be an efficient approach to enhance embodied presence.

The onset times that we obtained in Experiment 3 may furthermore be interesting to game developers and illusion researchers alike because they can be used to establish the minimally required duration of synchronous stroking or movement to establish a FBI. We demonstrated that participants need on average 33 s before they experience an illusion over the body that they see in front of them if a 3PP is used and that 95% of the participants experience the illusion within 95 s. However, it is important to note that these numbers come from individuals who have at least some experience with the illusion. Future research could investigate whether the onset times are similar in a truly virtual environment where gamers use their real body to control the movements of their avatar in a more natural and less repetitive manner. Kalckert and Ehrsson (2017) found that participansts were a bit faster to signal the RHI in an active induction condition (21 s) than with passive induction (24 s). In their study 95% of the participants felt the illusion within the first minute.

Although the present study contributed several new insights in the nature of illusory embodiment, several limitations may be noted. First, there is a remote possibility that tactile information was covertly activated in the movement condition. Although participants did not touch their necks in the movement condition and waved their hand up and down at a distance of approximately 10 cm from the neck, it is known that visuotactile neurons in parietal and premotor regions of the cortex respond to objects that loom in peripersonal space (Graziano et al., 1997, 1999). It should be noted however that these bimodal neurons are predominantly activated by objects that move towards the body, and much less so or not by objects that move away from the body (Canzoneri et al., 2012; Clery et al., 2015; Graziano & Cooke, 2006; Kandula et al., 2015), or that are on a trajectory that is not likely to impact the body (Huijsmans et al., 2020). Considering that the movements in the waving condition were parallel to the body it is questionable if these movements triggered covert tactile activations in this system.

A second limitation of the current study is that tactile stimulation in the stroking condition may be considered as an action effect that is known to enhance agency (Choi et al., 2016; Riemer et al., 2019; Wen et al., 2016) and that could strengthen the induced body illusion. However, the repetitive stroking movement was intransitive and no discrete goal or effect was obtained in executing the stroking. As such it is unlikely that the stroking condition generated more agency than the movement condition. This interpretation is furthermore supported by the fact that no significant differences were found in the strength of the FBI between conditions.

A third limitation is that no measure of agency was included in the current experiments. Previous research suggested that the experience of agency might enhance the strength of the ownership illusion (Ma & Hommel, 2015; Ma et al., 2017). In the design of the current study we controlled for potential differences in agency between conditions by comparing two methods that both employed active movements to induce the illusion. Although we cannot exclude the possibility that differences in agency may have existed between conditions, we reckon this to be unlikely considering the strong similarity in illusion strength between conditions and the matched design.

A final limitation is that the dependent measures in this study were mostly subjective, consisting of ratings on statements that captured the essence of the FBI and self-reported onset times of the FBI. Consequently, it could be argued that participants’ answers may not have objectively captured if they experienced the illusion and to what degree. It has to be noted though that the brief interviews after Experiment 2 provide us with some clues about who experienced the illusion and who did not and Experiment 3 even included extensive systematic interviews to identify participants who experienced the illusion with both induction methods. Moreover, we included several control questions in Experiment 3 to rule out the possibility that participants answered in a socially desirable manner about their experience of the FBI. Finally, measures about the time of illusion onset were repeated twice to increase their reliability.

In conclusion, the current study consistently found over multiple experiments that added synchronous sensory information does not increase the strength of the FBI, its speed of onset, or the probability for the illusion to occur. These findings are in line with the all-or-nothing principle that has been proposed to underlie (illusory) embodiment. Further research is necessary, however, to disentangle the interactions between self-reported agency and body illusion strength, and to validate the all-or-nothing principle in various conditions of illusory and impaired embodiment.

## Electronic supplementary material

Below is the link to the electronic supplementary material.

Supplementary file1 (DOCX 18 kb)

Supplementary file2 (DOCX 38 kb)

Supplementary file3 (DOCX 112 kb)

## Data Availability

Research data will be put on the DANS EASY archive and will be made available by the corresponding author upon reasonable request.

## Footnotes

1 Please note that the order in which the experiments are presented here differs from the order in which the experiments were conducted. We have decided to discuss the experiment that was originally conducted last as the third experiment for a better flow in the argumentation structure.

2 Exp 2: https://osf.io/yb3tu/?view_only=ba666ff6bc444f518214ee48456b3b38 Exp 3: https://osf.io/9yan2/?view_only=b3e01a1cfb084d99a010c112ab0cd1d4 Exp 4: https://osf.io/8ftd7/?view_only=3fe88538fd914c7d97f3717051bd027a

3 In addition we also had a dichotomous ‘yes’/’no’ scale for each statement for exploratory purposes. Participants found it hard to use this scale and preferred the visual analogue scale. This scale was therefore inspected but not analysed in Experiment 1, 2 and 4. In Experiment 3 it was not included at all.

4 Note that Bayes factors of 1 indicate that there is no evidence, Bayes factors between 1 and 3 are considered to be weak or anecdotal, Bayes factors between 3 and 10 are considered to be moderate and Bayes factors larger than 10 are considered to be strong. Wetzels, R., van Ravenzwaaij, D., & Wagenmakers, E. J. (2015). *Bayesian Analysis*. https://www.ejwagenmakers.com/2015/BayesianAnalysisEnclopedia.pdf.

5 Experiment 2 was preregistered when only half of the data for Experiment 1 were collected. The effect size was therefore based on preliminary analysis of 12 participants.

6 The difference scores for the questionnaire data indicate that the assumption of normality may be violated. However, there is no non-parametric alternative for the 2 × 2 repeated measures ANOVA that was used in the other experiments. We conducted linear mixed effects models because the model diagnostics for these models indicated that the assumptions for this test were met. In addition, we conducted the robust counterpart of the lme (rlmer from the package robustlmm) that can be used when assumptions are violated, to demonstrate that a potential violation of normality was no issue here. The robust analysis yielded similar t-values, supporting the results of the lme (see Table SOM3_1). The original lme analyses are reported here because there is more agreement on how to calculate p-values for this test.

7 This information could also be distilled from the interviews that were conducted at the end of Experiment 2. However, as the classification of participants was determined post-hoc based on less structured interviews it should be interpreted with caution. The probability analysis for Experiment 2 can be found in SOM3.
