# Supplementary material for: Availability of synchronous information in an additional sensory modality does not enhance the full body illusion
Source: Psychol Res. 2020 Jul 27;85(6):2291–312. doi: 10.1007/s00426-020-01396-z (PMC8357710; doi:10.1007/s00426-020-01396-z)
Supplement: Supplementary file 1 — Supplementary file1 (DOCX 18 kb) [file 426_2020_1396_MOESM1_ESM.docx]

**Supplementary Online Materials 1: Pressure pain measurement**

Article title: Availability of Synchronous Information in an Additional Sensory Modality Does Not Enhance the Full Body Illusion.

Journal name: Psychological Research

Authors: Lieke M.J. Swinkels, Harm Veling, Ap Dijksterhuis, Hein T. van Schie,

Corresponding author: Lieke M.J. Swinkels, Behavioural Science Institute, Radboud University, l.swinkels@bsi.ru.nl

In line with work by [Hänsel, Lenggenhager, von Känel, Curatolo, and Blanke (2011](#_ENREF_1)) who demonstrated increased pressure pain thresholds after a change in self-location and self-identification had been induced, we wanted to explore pressure pain thresholds in relation to the self-generated full body illusion (FBI). A digital algometer (Wagner instruments, FPX 25) was used to measure the pressure pain threshold before and after each movement block. The measurements were only completed for the movement blocks of Experiment 1 and not after the stroking blocks because we merely wanted to explore the pressure pain thresholds and we wanted to keep the number of measurements to a minimum for the participants. We hypothesized that participants would demonstrate increased pressure pain thresholds after completion of the synchronous movement blocks as compared to before these blocks were completed. No differences in pressure pain threshold were expected for the static movement blocks.

**Procedure**

At the beginning of the experiment participants were instructed to place their dominant hand on the table with the palm of the hand facing downwards. They were instructed to find a spot on their lower arm, just below the elbow (on the brachioradialis muscle) where they could feel pressure pain without having to press very hard. This spot was marked for the measurements that would be conducted later in the experiment. Before and after each completion of a movement block the participant completed 3 pressure pain measurements, resulting in a minimum of 12 measurements per participant. If one of the three measurements deviated more than 6N/ cm^2^ from the mean of the three measurements, the program asked for additional measurements. The pre-measures were completed before the head mounted display (HMD) was put on, the post-measures were completed while participants still had the HMD on their head. In both cases the experimenter guided the participant’s non-dominant hand to the spot that was marked at the beginning of the experiment. The non-dominant hand was chosen because participants used their dominant hand to make the stroking and waving movements. Making these movements could have tired their arm hereby affecting the amount of pressure they used during the measurements. Participants were instructed to press the algometer down on the marked location, while looking at either the wall in front of them (pre-measure) or at the body in front of them (post-measure), until the pressure became painful.

**Results**

A RM ANOVA with measurement (pre, post) and video condition (synchronous, static) as within subject factors was conducted on the average pain thresholds. This analysis revealed a trend for the main effect of measurement, *F*(1, 23) = 4.15, *p* = .053, *η^2^* = .15, indicating that participants on average report lower pain thresholds on the post-measure (*M* = 28.94, *SD* = 16.71) compared to the pre-measure (*M* = 31.70, *SD* = 19.93) regardless of the video condition they just completed. No significant main effect of video condition was obtained, *F*(1, 23) = .661, *p* = .425, nor a significant interaction effect, *F*(1, 23) = .609, *p* = .443.

**Discussion**

The pain measurements that were conducted in this experiment did not show the hypothesized increase in pressure pain threshold after completion of the synchronous movement blocks. It seems like the FBI was not associated with changes in pain perception in our experiment. However, we have reason to believe that these results may be due to a flaw in our procedure. This flaw relates to the marginally significant main effect of measurement. Already during data collection, we noticed that participants were clumsier with the algometer during the completion of the post-measure where they could only see their own body from a third person perspective as compared to the pre-measure where they were still able to see their own body in the periphery. We therefore cannot rule out that participants handled the algometer differently in the post-measures. A small difference in the angle or a difference in the speed with which the pressure is applied could affect the pain threshold. For future research we would recommend that the experimenter applies the pressure pain stimulus or use a device that automatically increases the pressure until participants press a button.

**References**

Hänsel, A., Lenggenhager, B., von Känel, R., Curatolo, M., & Blanke, O. (2011). Seeing and identifying with a virtual body decreases pain perception. *European Journal of Pain, 15*(8), 874-879. doi: <http://dx.doi.org/10.1016/j.ejpain.2011.03.013>
