# Supplementary material for: Availability of synchronous information in an additional sensory modality does not enhance the full body illusion
Source: Psychol Res. 2020 Jul 27;85(6):2291–312. doi: 10.1007/s00426-020-01396-z (PMC8357710; doi:10.1007/s00426-020-01396-z)
Supplement: Supplementary file 2 — Supplementary file2 (DOCX 38 kb) [file 426_2020_1396_MOESM2_ESM.docx]

**Supplementary Online Materials 2: Taste perception**

Article title: Availability of Synchronous Information in an Additional Sensory Modality Does Not Enhance the Full Body Illusion.

Journal name: Psychological Research

Authors: Lieke M.J. Swinkels, Harm Veling, Ap Dijksterhuis, Hein T. van Schie

Corresponding author: Lieke M.J. Swinkels, Behavioural Science Institute, Radboud University, l.swinkels@bsi.ru.nl

**Experiment 2**

In the second experiment we wanted to explore whether the consequences for the real body associated with body illusions that have been found in the domains of skin temperature (Moseley et al., 2008; Salomon, Lim, Pfeiffer, Gassert, & Blanke, 2013), pain perception (Hänsel, Lenggenhager, von Känel, Curatolo, & Blanke, 2011; Siedlecka, Klimza, Łukowska, & Wierzchoń, 2014) and tactile processing (Moseley et al., 2008; Swinkels, van Schie, Veling, ter Horst, & Dijksterhuis, 2020) would extend to the domain of taste perception. We hypothesized that participants would perceive the same grenadine solution as less sweet after completion of the synchronous conditions as compared to the static conditions.

**Materials**

In a practice block, participants were presented with three grenadine solutions (none, weak, strong) for which they had to rate the sweetness. The weak solution consisted of 10% grenadine and the strong solution consisted of 30% grenadine (see also: van der Wal & van Dillen, 2013). The solutions were served in quantities of 20 ml in red cups and were tasted using red straws to ensure that participants could not use vision to determine the strength of the solution. Each solution was scored on a scale ranging from 0 (not at all sweet) to 100 (very sweet). During the experimental taste session, the participants only tasted the strong solution.

**Procedure**

In the practice block the participants first received the solution without grenadine, followed by the weak and the strong solution. Participants were instructed to look at the wall in front of them and to consume the drinks all at once using the straw that was provided. Immediately after tasting a solution, participants provided a sweetness score which was logged on the computer by the experimenter. Participants were instructed to take a sip of water in between each tasting to cleanse their palate. Next they completed a block of the full body illusion (FBI) task. After each block, participants were presented with the same strong solution (30% grenadine) but were lead to believe that the presented drinks could differ in sweetness. For the static conditions a new static image of the participant holding the cup was created. Participants were instructed to focus on the body in front of them while tasting the drink using the straw. The drinks were scored as was practiced during the practice block.

**Statistical analysis**

A repeated measures ANOVA with video condition (sync, static) and induction method (movement, stroking) as within subject factors was conducted on the mean sweetness ratings to test whether the grenadine was rated as less sweet after the experimental synchronous condition compared to the static control condition. In addition, we used a linear mixed effects model approach using the lmer function of the lme4 package (version 1.1-12; Bates, Mächler, Bolker, & Walker, 2015) in R (R Core Team, 2015). Our model included a fixed intercept and a fixed effect for the factor video condition (coded using sum to zero contrasts). The repeated measures nature of the data was modeled by including a per-participant random adjustment to the fixed intercept (“random intercept”). To determine p-values we computed Type 3 bootstrapped Likelihood Ratio Tests (using 1000 simulations) as implemented in the mixed function of the package afex (Singmann, Bolker, Westfall, & Aust, 2017), which in turn called the function PBmodcomp of the package pbkrtest (Halekoh & Højsgaard, 2014)

**Results**

**Sweetness perception – Exploratory**

We conducted a repeated measures ANOVA with induction method (movement, stroking) and video condition (sync, static) on the sweetness ratings that were provided after each block (post-sweetness). We obtained a marginally significant effect of video condition on the sweetness ratings, *F*(1, 19) = 3.23, *p* = .088, *η^2^* = .15. The effect was in the predicted direction such that participants experienced the same drink as less sweet after completion of the synchronous condition (*M* = 76.72) compared to the static condition (*M* = 80.70). We did not find a significant main effect of induction method, *F*(1, 19) = 0.30, *p* = .589, nor a significant interaction effect, *F*(1, 19) = 0.0003, *p* = .987.

*Linear mixed effects model.* In addition, to further test whether the lemonade samples are experienced as less sweet after the synchronous experimental condition compared to the static control condition, we used a linear mixed effects model approach using the lmer function of the lme4 package (version 1.1-12; Bates et al., 2015) in R (R Core Team, 2015). The analysis showed that the perceived sweetness decreased significantly as a function of the video condition the participants completed, Estimate = 2.11(0.98), PBtest = 4.67, *p* = .032. Participants experienced the same drink as less sweet after completion of the synchronous experimental condition (*M* = 76.72) compared to the static control condition (*M* = 80.70).

**Experiment 4**

In Experiment 4 we tried to replicate the promising results of Experiment 2. The power calculations for Experiment 4 were based on the effect size obtained for the sweetness perception in Experiment 2 (see page 20 of the main text for more information). The materials and procedures used for Experiment 4 were the same as for Experiment 2 with one exception: in Experiment 4, participants only completed the self-generated movement condition.

**Results**

**Sweetness perception – confirmatory**

*Repeated measures ANOVA.* We conducted a repeated measures ANOVA with video condition (sync, static) on the sweetness ratings that were provided after each block (post-sweetness). Contrary to our hypothesis we did not obtain a significant main effect of video condition on the sweetness ratings, *F*(1, 49) = 0.23, *p* = .631.

*Linear mixed effects model.* In addition, to further test whether the lemonade samples are experienced as less sweet after the synchronous experimental condition compared to the static control condition, we used a linear mixed effects model approach using the lmer function of the lme4 package (version 1.1-12; Bates et al., 2015) in R (R Core Team, 2015). The analysis shows that the perceived sweetness did not decrease significantly as a function of the video condition the participants completed, Estimate = -0.50(0.70), PBtest = 0.52, *p* = .507. Participants experienced the same drinks as equally sweet after completion of the synchronous experimental condition (*M* = 75.45) and the static control condition (*M* = 74.45).

*Exploratory repeated measures ANOVA.* Contrary to our predictions we found no significant main effect of video condition on perceived sweetness. However, it could be the case that the perceived sweetness decreased for both video conditions compared to baseline. This might indicate that mere exposure to an external body without illusory embodiment may be enough to reduce the experienced sweetness of the drinks. Since there was no difference between the two video conditions we collapsed to conditions and calculated the mean perceived sweetness. We ran a RM ANOVA with measurement (pre-measure, post-measure) as within subject factor. This analysis indicated a significant main effect of measurement, *F*(1, 49) = 9.12, *p* = .003, η_p_^2^ = .17. On average participants experienced the grenadine solution as sweeter during the baseline measurement (*M* = 80.08, *SD* = 14.04) than during the post measure (*M* = 75.20, *SD* = 13.75).

**Discussion**

The promising findings of Experiment 2 did not replicate in Experiment 4. Contrary to other experiments that found costs for the real body related to the FBI in the domain of skin temperature (Moseley et al., 2008; Salomon et al., 2013), pain perception (Hänsel et al., 2011; Siedlecka et al., 2014) and tactile processing (Moseley et al., 2008; Swinkels, van Schie, Veling, ter Horst, & Dijksterhuis, 2020), we did not find an effect on sweetness perception. As Experiment 4 was better powered than Experiment 2, the findings in Experiment 2 may reflect a false positive result.
 Usually, costs for the real body are rather subtle. It may therefore be the case that our 100-point self-report scale just was not sensitive enough to pick up on changes in taste perception. However, the effects may also be of a more general nature. The exploratory repeated measures ANOVA in which we compared the baseline sweetness perception to the post measure collapsed over video conditions suggests that mere exposure to an external body without illusory embodiment may be enough to reduce the experienced sweetness of the drinks. This is in line with findings by Branton et al. (2014) who demonstrated reduced food intake after participants engaged in screen based activity. Participants who engaged in video gameplay for 30 minutes demonstrated reduced food intake afterwards compared to a control group that engaged in 30 minutes of non-screen activities. The presented findings on taste perception in Experiment 3 suggest that comparable effects of screen activity on the appeal of food and drinks may extend to conditions in which individuals use HMDs. Further research is necessary to investigate the what factors (e.g. the attractiveness or appeal of visual stimuli or the tendency of individuals to become absorbed) that drive these effects.

**References**

Bates, D., Mächler, M., Bolker, B., & Walker, S. (2015). Fitting linear mixed-effects models using lme4. *2015, 67*(1), 48. doi:10.18637/jss.v067.i01

Branton, A., Akhavan, T., Gladanac, B., Pollard, D., Welch, J., Rossiter, M., & Bellissimo, N. (2014). Pre-meal video game playing and a glucose preload suppress food intake in normal weight boys. *Appetite, 83*, 256-262. doi:10.1016/j.appet.2014.08.024

Halekoh, U., & Højsgaard, S. (2014). A Kenward-Roger approximation and parametric bootstrap methods for tests in linear mixed models - The R package pbkrtest. *2014, 59*(9), 32. doi:10.18637/jss.v059.i09

Hänsel, A., Lenggenhager, B., von Känel, R., Curatolo, M., & Blanke, O. (2011). Seeing and identifying with a virtual body decreases pain perception. *European Journal of Pain, 15*(8), 874-879. doi:http://dx.doi.org/10.1016/j.ejpain.2011.03.013

Moseley, G. L., Olthof, N., Venema, A., Don, S., Wijers, M., Gallace, A., & Spence, C. (2008). Psychologically induced cooling of a specific body part caused by the illusory ownership of an artificial counterpart. *Proceedings of the National Academy of Sciences, 105*(35), 13169-13173. doi:10.1073/pnas.0803768105

R Core Team. (2015). R: A language and environment for statistical computing: R Foundation for Statistical Computing. Retrieved from https://www.R-project.org

Salomon, R., Lim, M., Pfeiffer, C., Gassert, R., & Blanke, O. (2013). Full body illusion is associated with widespread skin temperature reduction. *Frontiers in Behavioral Neuroscience, 7*. doi:10.3389/fnbeh.2013.00065

Siedlecka, M., Klimza, A., Łukowska, M., & Wierzchoń, M. (2014). Rubber hand illusion reduces discomfort caused by cold stimulus. *PLOS ONE, 9*(10), e109909. doi:10.1371/journal.pone.0109909

Singmann, H., Bolker, B., Westfall, J., & Aust, F. (2017). afex: Analysis of factorial experiments (Version 0.18-0). Retrieved from https://CRAN.R-project.org/package=afex

Swinkels, L. M. J., van Schie, H. T., Veling, H., ter Horst, A. C., & Dijksterhuis, A. (2020). The self-generated full body illusion is accompanied by impaired detection of somatosensory stimuli. *Acta Psychologica, 203*, 102987. doi:https://doi.org/10.1016/j.actpsy.2019.102987

van der Wal, R. C., & van Dillen, L. F. (2013). Leaving a Flat Taste in Your Mouth. *Psychological Science, 24*(7), 1277-1284. doi:doi:10.1177/0956797612471953
