# Supplementary material for: Availability of synchronous information in an additional sensory modality does not enhance the full body illusion
Source: Psychol Res. 2020 Jul 27;85(6):2291–312. doi: 10.1007/s00426-020-01396-z (PMC8357710; doi:10.1007/s00426-020-01396-z)
Supplement: Supplementary file 3 — Supplementary file3 (DOCX 112 kb) [file 426_2020_1396_MOESM3_ESM.docx]

**Supplementary Online Materials 3**

Article title: Availability of Synchronous Information in an Additional Sensory Modality Does Not Enhance the Full Body Illusion.

Journal name: Psychological Research

Authors: Lieke M.J. Swinkels, Harm Veling, Ap Dijksterhuis, Hein T. van Schie

Corresponding author: Lieke M.J. Swinkels, Behavioural Science Institute, Radboud University, [l.swinkels@bsi.ru.nl](mailto:l.swinkels@bsi.ru.nl)

**Results of robust linear mixed effects model**

In addition to the regular linear mixed effects model analysis a robust linear mixed effects model analysis was conducted (Koller, 2016) because there were some concerns about the normality of the data. To obtain p-values for the robust analysis we used Satterthwaite approximations of degrees of freedom as described by (Geniole et al., 2019). See Table SOM3_1 for the results of the robust analysis and a comparison with the original analysis.

**Table SOM3_1.** Full statistic results of the original and robust linear mixed effects model analyses conducted on the illusion statements in Experiment 3. The original and the robust analysis yielded the same conclusions except for one instance that is indicated in red.

|  | Original analysis | | | | Robust analysis | | | |
| --- | --- | --- | --- | --- | --- | --- | --- | --- |
|  | Estimate | SE | *t* | *p* | Estimate | SE | *t* | *p* |
| S1 |  |  |  |  |  |  |  |  |
| inductionMethod | -1.35 | 1.31 | -1.03 | .293 | -1.34 | 1.37 | -0.98 | .329 |
| videoCondition | -17.44 | 1.31 | -13.32 | <.001 | -18.03 | 1.37 | -13.16 | <.001 |
| interaction | -1.84 | 1.31 | -1.40 | .175 | -1.62 | 1.37 | -1.19 | .237 |
| S2 |  |  |  |  |  |  |  |  |
| inductionMethod | -0.09 | 1.26 | -0.07 | .937 | -0.04 | 1.32 | -0.03 | .977 |
| videoCondition | -15.84 | 1.26 | -12.55 | <.001 | -15.94 | 1.32 | -12.12 | <.001 |
| interaction | -0.39 | 1.26 | 0.31 | .760 | 0.22 | 1.32 | 0.17 | .867 |
| S3 |  |  |  |  |  |  |  |  |
| inductionMethod | -2.19 | 1.12 | -1.96 | .063 | -2.35 | 1.09 | -2.16 | .032 |
| videoCondition | -10.88 | 1.12 | -9.74 | <.001 | -10.32 | 1.09 | -9.52 | <.001 |
| interaction | 0.28 | 1.12 | 0.25 | .831 | 0.56 | 1.09 | 0.51 | .609 |
| C1 |  |  |  |  |  |  |  |  |
| inductionMethod | -1.28 | 0.86 | -1.49 | .132 | -1.14 | 0.79 | -1.44 | .151 |
| videoCondition | -2.40 | 0.86 | -2.80 | .012 | -2.46 | 0.79 | -3.11 | .002 |
| interaction | 0.43 | 0.86 | 0.50 | .625 | 0.22 | 0.79 | 0.28 | .777 |
| C2 |  |  |  |  |  |  |  |  |
| inductionMethod | -2.82 | 1.00 | -2.83 | .004 | -3.05 | 0.84 | -3.65 | <.001 |
| videoCondition | -1.33 | 1.00 | -1.33 | .204 | -1.63 | 0.84 | -1.95 | .052 |
| interaction | 1.64 | 1.00 | 1.64 | .097 | 1.46 | 0.84 | 1.74 | .083 |
| C3 |  |  |  |  |  |  |  |  |
| inductionMethod | -1.71 | 1.06 | -1.63 | .104 | -1.97 | 1.03 | -1.91 | .057 |
| videoCondition | -3.35 | 1.06 | -3.17 | .003 | -3.20 | 1.03 | -3.10 | .002 |
| interaction | -.017 | 1.06 | -0.16 | .887 | 0.25 | 1.03 | 0.24 | .808 |
| Suggestibility |  |  |  |  |  |  |  |  |
| inductionMethod | -1.57 | 0.55 | -2.87 | .002 | -1.57 | 0.58 | -2.70 | .007 |
| videoCondition | -8.54 | 0.55 | -15.61 | <.001 | -8.99 | 0.58 | -15.44 | <.001 |
| statement | 4.31 | 0.55 | 7.88 | <.001 | 4.62 | 0.58 | 7.93 | <.001 |
| inMeth * vidCon | 0.12 | 0.55 | 0.22 | .818 | 0.22 | 0.58 | 0.39 | .699 |
| inMeth * stat | -0.37 | 0.55 | -0.67 | .484 | -0.59 | 0.58 | -1.02 | .310 |
| vidCon * stat | 6.18 | 0.55 | 11.29 | <.001 | 6.37 | 0.58 | 10.94 | <.001 |
| 3-way interaction | 0.51 | 0.55 | 0.93 | .331 | 0.57 | 0.58 | 0.98 | .327 |

**Probability of experiencing the FBI**

As for Experiment 3, we explored whether participants would be more likely to report the illusion for one of the two induction methods in Experiment 2. Brief talks with the participants at the end of the experiment provided us with some information on whether an illusion was experienced for one of the two methods. However, these numbers should be interpreted with caution as these classifications were made based on a post-hoc interpretation of the interview notes. In line with Experiment 3, we decided to classify a participant as experiencing the illusion for a specific induction method, if 1) they had a higher average score on the illusion statements in the synchronous condition compared to the static control condition for this method and 2) they indicated at the end of the experiment to have experienced the illusion for this method.
 Of the final number of 20 participants in Experiment 2, 7 participants experienced the illusion for both induction methods, 2 participants only experienced the illusion for the movement method, 4 participants only experienced the illusion for the stroking method and 7 participants did not experience the illusion at all. In percentages this would mean that 35% percent of the participants experienced the illusion for both methods and 65% of the participants would have experienced the illusion for at least one method. 35% of the participants indicated to not have experienced the illusion at all.
 One participant has been classified as not experiencing the illusion because the notes from the interview were missing for this participant. Based on the illusion scores this participant would have experienced the illusion. That is, this participant had scores well above the midpoint of the scale for the synchronous conditions for both methods and scores well below the midpoint of the scale for the static conditions for both methods. In this case not 7 but 8 participants would have experienced the illusion for both induction methods, and not 7 but 6 participants would not have experienced the illusion at all. This would mean that 40% of the participants experienced the illusion for both methods, 70% of the participants experienced the illusion for at least one method and 30% of the participants did not experience the illusion at all.
 To test whether participants would be more likely to report the illusion for one of the two methods, we again first tried to run a generalized linear mixed model with the binary outcome variable illusion onset (yes, no), a fixed intercept, a random intercept for participant and induction method as a fixed factor. However as for Experiment 3, the model failed to converge. We therefore conducted a RM logistic regression analysis in SPSS instead. This analysis indicated that participants report the illusion just as often for the self-generated stroking method as for the self-generated movement method, OR = 0.67, 95% CI = [-0.55, 1.36], Wald Chi-square = 0.68, p = .409.

**Histograms of raw illusion scores**

The histograms of the raw illusion scores suggest that the rather low average illusion scores that were obtained in our experiments may have been due to a bimodal distribution of the data. Some participants reported a rather strong illusion experience whereas other participants reported not to experience an illusion at all. In the supplementary figures below we present the bimodal distributions that arose in the various levels of analysis. The bimodal distribution is visible when you collapse across experiments, induction methods and items (Figure SOM3_1), when you collapse across experiments and induction methods (Figure SOM3_2), but also when you look at the distributions separately for each experiment, condition and item (Figures SOM3_3 – SOM3_6).

**Figure SOM3_1** Histogram of the raw illusion scores collapsed across experiments, induction methods and items (S1 and S2).

**Figure SOM3_2** Histograms of the raw illusion scores collapsed across experiment and induction methods, presented separately for the two items.


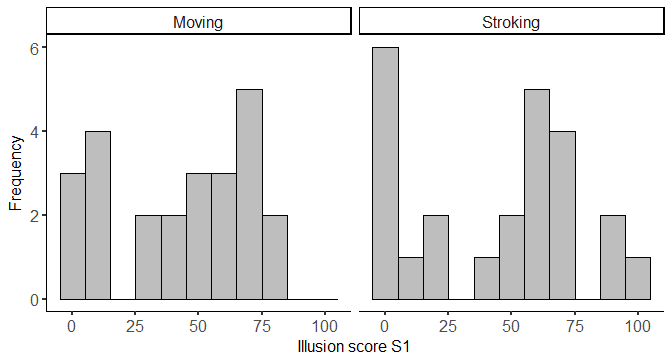


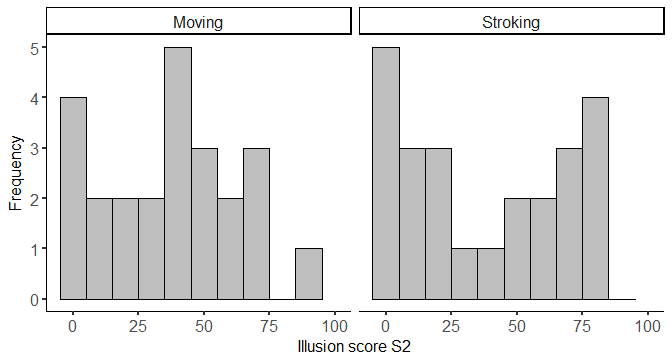


**Figure SOM3_3** Histograms of the raw illusion scores obtained for S1 and S2 in Experiment 1. The histograms are depicted separately for the two induction methods.


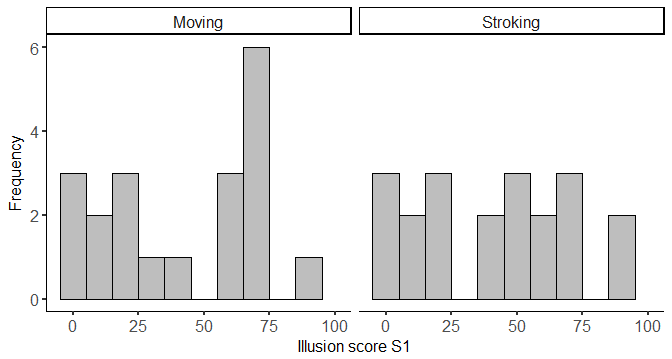

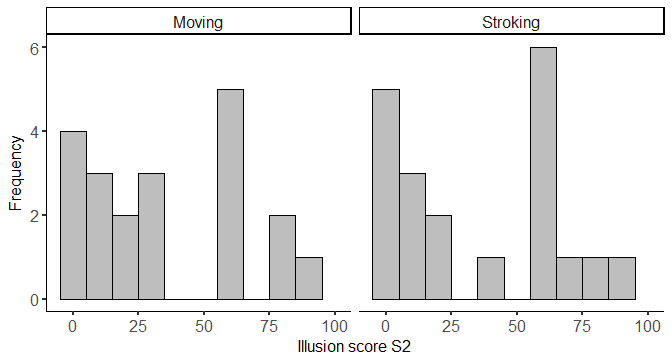


**Figure SOM3_4** Histograms of the raw illusion scores obtained for S1 and S2 in Experiment 2. The histograms are depicted separately for the two induction methods.


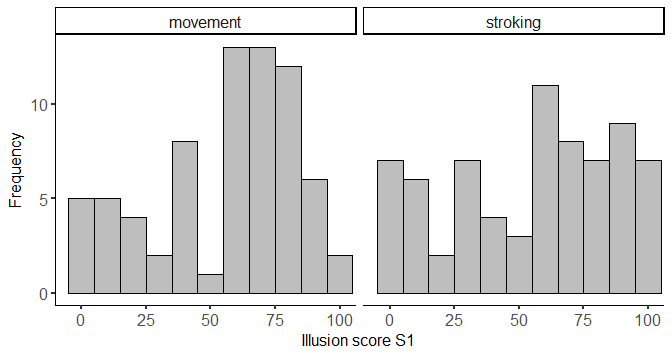


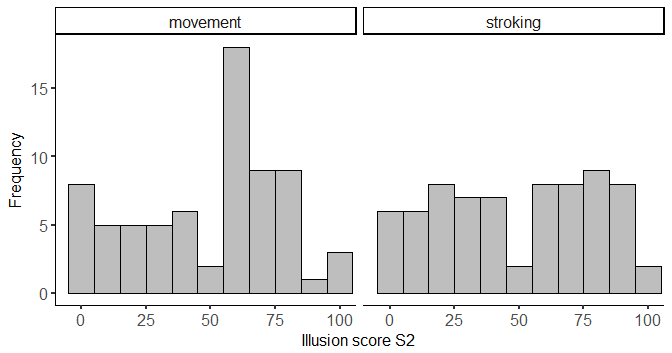


**Figure SOM3_5** Histograms of the raw illusion scores obtained for S1 and S2 in Experiment 3. The histograms are depicted separately for the two induction methods.


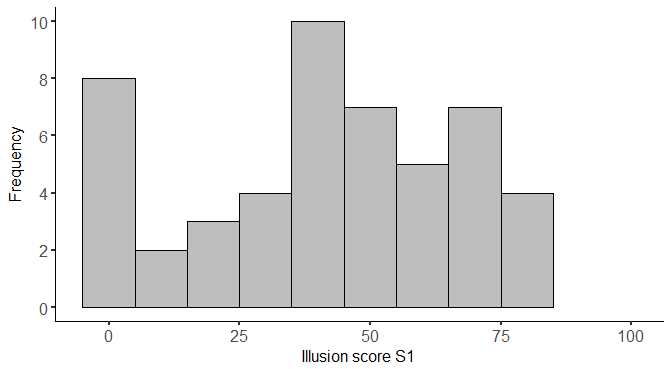

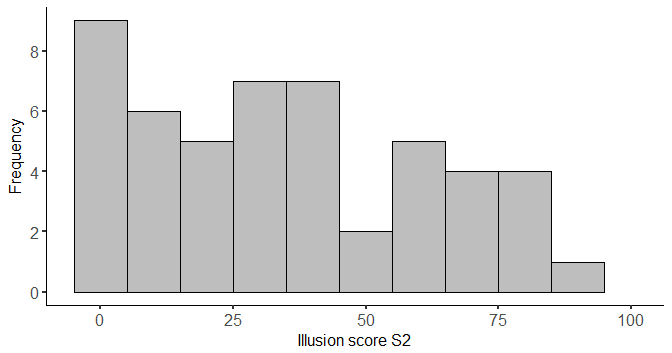


**Figure SOM3_6** Histograms of the raw illusion scores obtained for S1 and S2 in Experiment 4.

**References**

Geniole, S. N., Proietti, V., Bird, B. M., Ortiz, T. L., Bonin, P. L., Goldfarb, B., Watson, N. V., & Carré, J. M. (2019, 2019/05/29). Testosterone reduces the threat premium in competitive resource division. *Proceedings of the Royal Society B: Biological Sciences, 286*(1903), 20190720. https://doi.org/10.1098/rspb.2019.0720

Koller, M. (2016, 2016-12-06). robustlmm: An R Package for Robust Estimation of Linear Mixed-Effects Models [robust statistics; mixed-effects model; hierarchical model; ANOVA; R; crossed; random effect]. *2016, 75*(6), 24. https://doi.org/10.18637/jss.v075.i06
